# Single-cell RNA sequencing identifies molecular biomarkers predicting late progression to CDK4/6 inhibition in patients with HR+/HER2- metastatic breast cancer

**DOI:** 10.1186/s12943-025-02226-9

**Published:** 2025-02-15

**Authors:** Linjie Luo, Peng Yang, Sofia Mastoraki, Xiayu Rao, Yan Wang, Nicole M. Kettner, Akshara Singareeka Raghavendra, Debasish Tripathy, Senthil Damodaran, Kelly K. Hunt, Jing Wang, Ziyi Li, Khandan Keyomarsi

**Affiliations:** 1 Department of Experimental Radiation Oncology, The University of Texas MD Anderson Cancer Center, Houston, TX USA; 2 Department of Statistics, Rice University, Houston, TX USA; 3 Department of Biostatistics, The University of Texas MD Anderson Cancer Center, Houston, TX USA; 4 Department of Bioinformatics and Computational Biology, The University of Texas MD Anderson Cancer Center, Houston, TX USA; 5 Department of Breast Medical Oncology, Division of Cancer Medicine, The University of MD Anderson Cancer Center, Houston, TX USA; 6 Department of Breast Surgical Oncology, The University of Texas MD Anderson Cancer Center, Houston, TX USA

**Keywords:** CDK4/6 inhibitor, Drug resistance, Metastatic breast cancer, Outcome prediction, Predictive biomarker, Single-cell RNA-sequencing, Target therapy, Transcriptomics, Tumor microenvironment, Tumor-infiltrating lymphocytes

## Abstract

**Background:**

Cyclin-dependent kinase 4/6 inhibitors (CDK4/6is) in combination with endocrine therapy are the standard treatment for patients with hormone receptor–positive, HER2–negative metastatic breast cancer (mBC). Despite the efficacy of CDK4/6is, intrinsic resistance occurs in approximately one-third of patients, highlighting the need for reliable predictive biomarkers.

**Methods:**

Single-cell RNA sequencing analyzed metastatic tumors from HR+/HER2- mBC patients pre-CDK4/6i treatment at baseline (BL) and/or at disease progression. BL samples were from CDK4/6i responders (median progression-free survival [mPFS] = 25.5 months), while progressors were categorized as early-progressors (EP, mPFS = 3 months) and late-progressors (LP, mPFS = 11 months). Metastatic sites included liver, pleural effusions, ascites, and bone. InferCNV distinguished tumor cells, and functional analysis utilized the Molecular Signatures Database.

**Results:**

LP tumors displayed enhanced Myc, EMT, TNF-α, and inflammatory pathways compared to those EP tumors. Samples from BL and LP responders showed increased tumor-infiltrating CD8^+^ T cells and natural killer (NK) cells compared to EP non-responders. Notably, despite a high frequency of CD8^+^ T cells in responding tumors, a functional analysis revealed significant upregulation of genes associated with stress and apoptosis in proliferative CD4^+^ and CD8^+^ T cells in BL tumors compared to in EP and LP tumors. These genes, including *HSP90* and *HSPA8*, are linked to resistance to PD1/PD-L1 immune checkpoint inhibitors. A ligand-receptor analysis showed enhanced interactions associated with inhibitory T-cell proliferation (SPP1-CD44) and suppression of immune activity (MDK-NCL) in LP tumors. Longitudinal biopsies consistently revealed dynamic NK cell expansion and enhanced cytotoxic T cell activity, alongside upregulation of immune activity inhibition, in LP tumors compared to in BL tumors. Notably, the predictive biomarker panel from BL tumor cells was validated in 2 independent cohorts, where it consistently predicted a significant improvement in mPFS duration in signature-high versus -low groups.

**Conclusion:**

This study underscores the significance of molecular biomarkers in predicting clinical outcomes to CDK4/6i. Tumor-infiltration CD8^+^ T and NK cells may also serve as baseline predictors. These insights pave the way for optimizing therapeutic strategies based on microenvironment-specific changes, providing a personalized and effective approach for managing HR+/HER2- mBC and improving patient outcomes.

**Supplementary Information:**

The online version contains supplementary material available at 10.1186/s12943-025-02226-9.

## Background

Breast cancer (BC) is the most common non-cutaneous cancer among women worldwide [1], with hormone receptor–positive (HR+), HER2–negative (HER2-) subtypes accounting for over 70% of diagnoses [2]. Approximately 30% of patients with early-stage disease eventually experience relapse with metastatic disease [3].

For patients with HR+/HER2- metastatic BC (mBC), the combination of cyclin-dependent kinase 4 and 6 inhibitors (CDK4/6is) with endocrine therapy has become the standard of care [4]. FDA-approved CDK4/6is, including palbociclib [5–7], ribociclib [8–10], and abemaciclib [11–14], have consistently demonstrated prolonged progression-free survival (PFS) compared to endocrine therapy alone. However, despite these advancements, approximately one-third of patients exhibit early (intrinsic) resistance to treatment within 6 months [15], with early-onset disease progression. Additionally, nearly all patients eventually develop acquired resistance, characterized by late-onset progression after an initial response and a period of disease stability [16]. These resistance mechanisms present a significant clinical challenge, emphasizing the need for deeper molecular insights and predictive biomarkers to guide treatment decisions.

Current efforts to identify biomarkers of response to CDK4/6i aim to enable a more personalized approach to treating patients with HR+/HER2- mBC. However, our understanding of the biological bases for intrinsic and acquired resistance remains incomplete, and there is no consensus on the optimal biomarkers for predicting CDK4/6i response. Proposed predictive factors include ERα and PR status [17], Rb mutation [5, 17–19], FAT1 loss of function [20], intrinsic molecular classification [17, 21, 22], CDK6 [23] and CCNE1 overexpression [24, 25], FGFR amplification [17], and PI3K/AKT/mTOR pathway activation [26]. Most studies have focused on genetic alterations, but challenges remain, including the limited assessment of target genes, which results in a significant proportion of undefined genetic alterations [27]. Additionally, gene expression profiles are still incompletely understood and under investigation.

Several unresolved questions persist. First, current studies have not thoroughly delineated the distinct mechanisms underlying early and late progression with CDK4/6is, and precise biomarkers to differentiate early progressors (EP) from late progressors (LP) are lacking. Second, most biomarker studies have concentrated on biopsies from primary tumors, with limited research examining metastatic sites across various anatomical locations. Intra-tumor and extra-tumor heterogeneity further complicates the identification of reliable biomarkers. The tumor microenvironment (TME) consists of diverse tumor and non-tumor cells, including immune and stromal cells, that influence tumor behavior and treatment response. Therefore, traditional bulk sequencing data may not fully capture the complexity within the tumor due to its low resolution and inability to distinguish the molecular profiles of individual cells. Addressing these knowledge gaps could enhance our understanding of CDK4/6i resistance and improve the identification of robust predictive biomarkers in mBC treatment.

The results of several studies suggest that, apart from their direct effect on tumor cells, CDK4/6is influence immune cells in the TME [28–30], including the suppression of regulatory T cell proliferation and enhanced activation of tumor-infiltrating lymphocytes (TILs) [31]. Dysregulation of these immune pathways may contribute to CDK4/6i resistance [29, 32]. Furthermore, the diverse cellular phenotypes and dynamic interactions within the TME create specific conditions in metastases across different organs, potentially affecting responses to CDK4/6i treatment and the identification of predictive biomarkers.

In this study, we employed single-cell RNA sequencing (scRNA-seq) to address key challenges in understanding CDK4/6i resistance in patients with HR+/HER2- mBC. By analyzing metastatic biopsies, both prior to and during tumor progression on CDK4/6is, scRNA-seq allowed us to unravel the intricate landscape of the TME and the interactions between tumor and immune cells. The single cell transcriptomic approach also enabled us to identify distinct gene expression profiles in tumor and non-tumor cells, providing insights into the mechanisms that drive early versus late disease progression on CDK4/6is. We validated these findings in 2 patient cohorts, underscoring the robustness and clinical relevance of our results. Our study reveals predictive molecular and cellular biomarkers that could guide treatment decisions, enabling a more personalized approach for patients with HR+/HER2- mBC.

## Methods

### Patient samples collection

Tumor biopsy samples were collected from patients with HR+/HER2- mBC treated at the University of Texas MD Anderson Cancer Center under the Institutional Review Board (IRB)-approved protocol. Eligible patients included those aged 18 years or older, diagnosed with HR+ (ER+ or PR+ ≥ 1% staining in primary or recurrent tumors) BC between January 1, 1997, and October 31, 2024, and either screened for mBC or suspected but not yet diagnosed. All participants signed informed consent. Clinical data were sourced from electronic medical records (OneConnect and Care Everywhere) and a prospectively maintained Breast Cancer Management System, managed by the Breast Medical Oncology Department from MD Anderson Cancer Center.

**Fig. 1:**
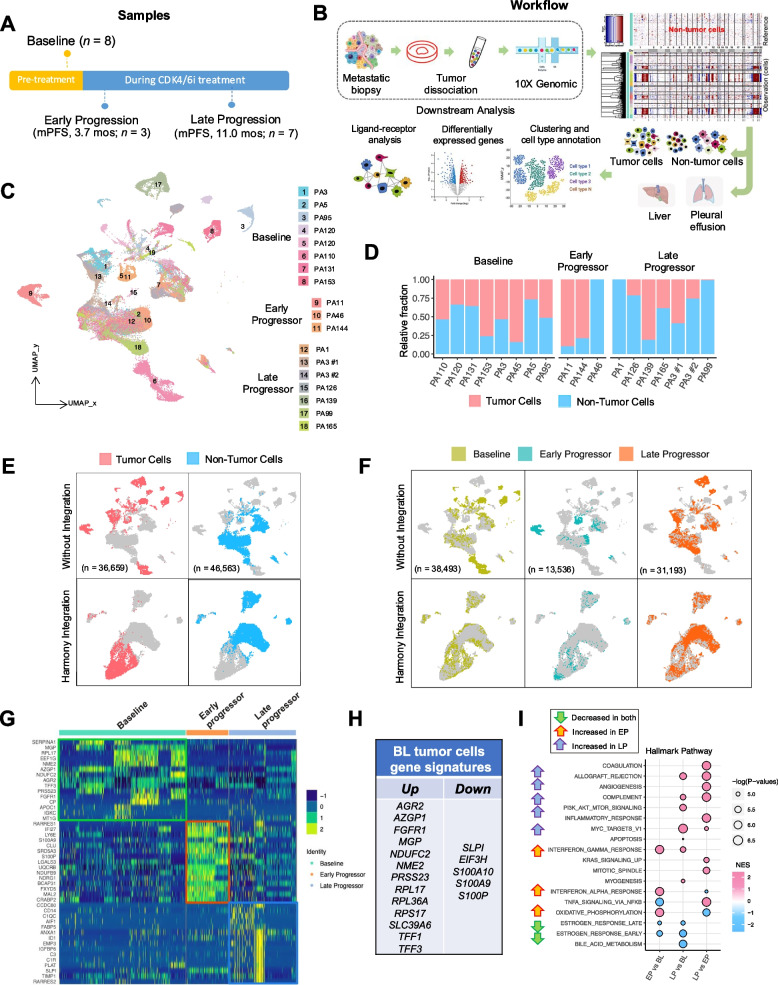
Identification of tumor and non-tumor cells. **A** Schematic depicting the study design, including 8 BL samples, 3 EP samples, and 7 LP samples from patients with HR+/HER2- mBC on CDK4/6i treatment. The cutoff for EP versus LP samples was a PFS duration of 6 months. **B** Study workflow illustrating sample processing, tumor cell identification (inferred by the presence of copy number aberrations using InferCNV), and downstream analysis. **C** UMAP plot of 18 samples in the embedding space. **D** Bar plot showing the relative fraction of tumor versus non-tumor cells for each sample, stratified by different sample statuses (BL, EP, and LP). **E** UMAP plot of tumor cells (left, pink) versus non-tumor cells (right, blue) without (top) or with (bottom) Harmony integration. **F** UMAP plot of cells, stratified by different sample statuses (BL, EP, and LP). **G** Heatmap displaying the expression of the top 15 differentially expressed genes, stratified by different sample statuses (BL, EP, and LP). **H** Predictive biomarker panels generated from the top differentially expressed genes from BL tumor cells. **I** Hallmark pathway analysis in tumor cells. The dot size represents the percentage of cells with the expression of certain genes under each sample status

In the scRNA-seq study, tumor biopsy samples were obtained from real-world patients with HR+/HER2- mBC before initiating CDK4/6i treatment (baseline [BL]), as well as at the time of disease progression during treatment. EP and LP tumors were defined using a cutoff of 6 months based on previous studies [16, 26]. Ultimately, we obtained 8 BL samples, 3 EP samples, and 7 LP samples (Fig. 1A). In this cohort, 75% of BL samples were from patients who received first-line treatment with CDK4/6i plus letrozole, while 25% of BL samples were from patients who received CDK4/6i plus fulvestrant as second-line treatment (Table 1). All EP samples were from patients who received palbociclib plus fulvestrant. Among the LP samples, 43% were collected from patients who received first-line treatment with palbociclib plus an aromatase inhibitor, while the remaining patients received palbociclib plus fulvestrant or ZN-c5 as second-line treatment (Table 1). Our sample collection of metastatic sites included 6 pleural effusions, 9 liver tumor samples, 2 ascites samples, and 1 pelvic bone sample (Table 1). Furthermore, 1 patient provided samples at multiple time points, including 1 BL sample and 2 LP samples collected at distinct time points during the progression of their disease. Three BL samples were collected from patients who were still undergoing treatment. PFS duration data were obtained from all participants, with the data cutoff in October 2024. All tissues were dissociated, and single-cell suspensions were prepared according to the manufacturer’s protocol.

**Table 1:** scRNA-seq sample information

Timepoint	Sample ID	PFS (months)	Metastatic Site	CDK4/6i	ET agent
Baseline (*n* = 8)	PA3	11	Liver	Palbociclib	Fulvestrant
	PA5	38	Pleural effusion	Palbociclib	Letrozole
	PA45	23	Liver	Palbociclib	Fulvestrant
	PA95	12^a^	Liver	Palbociclib	Letrozole
	PA110	31^b^	Pleural effusion	Palbociclib	Letrozole
	PA120	33^b^	Liver	Palbociclib	Letrozole
	PA131	28^b^	Liver	Abemaciclib	Letrozole
	PA153	18	Liver	Palbociclib	Letrozole
Early Progressor (*n* = 3)	PA46	5	Pleural effusion	Palbociclib	Fulvestrant
	PA11	3	Pleural effusion	Palbociclib	Fulvestrant
	PA144	3	Liver	Palbociclib	Fulvestrant
Late Progressor (*n* = 7)	PA1	13	Pleural effusion	Palbociclib	Fulvestrant
	PA3 #1	11	Ascites	Palbociclib	Fulvestrant
	PA3 #2	11	Ascites	Palbociclib	Fulvestrant
	PA126	9	Liver	Palbociclib	ZN-c5
	PA139	8	Liver	Palbociclib	Letrozole
	PA99	9	Pleural effusion	Palbociclib	Anastrozole
	PA165	37	Pelvic bone	Palbociclib	Anastrozole

^a^lost to follow-up

^b^still under treatment

In the validation study, we collected 89 BL samples from HR+/HER2- mBC patients at the University of Texas MD Anderson Cancer Center, including 35 fresh tissue biopsies and 54 formalin-fixed, paraffin-embedded (FFPE) samples (Table 2). In this cohort, 65% of patients received first-line treatment with CDK4/6i plus an aromatase inhibitor, while the remaining patients received CDK4/6i plus fulvestrant or other selective estrogen receptor degraders as second-line treatment. The majority of biopsies were from metastatic tissues, including bone, liver, and pleural effusion (Table 2). Most samples were from White patients (fresh tissue: 63%; FFPE: 72%), followed by Hispanic patients (fresh tissue: 17%; FFPE: 20%) (Table 2). PFS duration data were obtained from all participants.

**Table 2:** Charateristics of Patients in the MD Anderson Cohort

Parameter	N (%)		
	**Fresh tissue**	**FFPE**	**Master Database**
	(***n*** = 35)	(*n* = 54)	(*n* = 351)
**Mean age (range), y**	57.2 (28–88)	51.8 (29–79)	50.7 (19–85)
**Race/ethnicity**			
White	22 (62.8%)	39 (72.2%)	257 (73.2%)
Hispanic	6 (17.1%)	11 (20.4%)	39 (11.1%)
Black	2 (5.7%)	2 (3.7%)	29 (8.3%)
Asian	3 (8.6%)	0	20 (5.7%)
**CDK4/6i course**			
Palbociclib	24 (68.6%)	54 (100%)	257 (73.2%)
Abemaciclib	5 (14.3%)	0	42 (12.0%)
Ribociclib	6 (17.1%)	0	52 (14.8%)
**Endocrine therapy course**			
Aromatase inhibitor	23 (65.7%)	36 (66.7%)	240 (68.4%)
Fulvestrant	10 (28.6%)	16 (29.6%)	104 (29.6%)
Others	2 (5.7%)	2 (3.7%)	
**Mean duration of CDK4/6i + ET treatment, month (range), m**	19.4 (1–62)	15.3 (0.4–53)	23.8 (0.1–117.4)
**Source of biopsy**			**Site of metastasis**
Breast	0	12 (22.2%)	0
Bone	15 (42.9%)	18 (33.3%)	233 (66.4%)
Liver	8 (22.9%)	15 (27.8%)	62 (17.7%)
Pleural effusion	6 (17.1%)	2 (3.7%)	18 (5.1%)
Lung	1 (2.9%)	0	38 (10.8%)
Others	5 (14.3%)	7 (12.9%)	0

The master database from the University of Texas MD Anderson Cancer Center comprises real-world data on HR+/HER2- mBC patients treated with CDK4/6is between June 1, 2013, and October 23, 2024. Eligible patients were aged 18 or older, diagnosed with HR+ (ER+ or PR+ ≥ 1% staining in primary or recurrent tumors) BC, and either screened for or suspected of having mBC. Patients lost to follow-up were excluded. PFS was calculated from the start of CDK4/6i plus endocrine therapy to disease progression or the most recent follow-up. Both scRNA-seq and bulk RNA-seq data (from FFPE and fresh tissue in the MD Anderson cohort) were generated from patients in the master database. 

### Sample preparation for scRNA-seq

To make the dissociation solution, collagenase A (Sigma, 11,088,793,001) was dissolved in a 75% (vol/vol) DMEM F12/HEPES medium (Gibco, 113,300) and 25% (vol/vol) BSA fraction V (Gibco, 15,260,037) to achieve a final concentration of 1 mg/mL. To prepare viable single-cell suspensions, fresh tumors were placed in a 10-cm dish containing 5 mL of dissociation solution, minced into 1 mm^3^ pieces using scalpels, transferred to a 50-mL conical tube with 30 mL of dissociation solution, and incubated at 37 °C in a rotating hybridization oven for 20–25 min. The tissue suspension was filtered through a 70-μm strainer, with a syringe plunger flange used to grind any remaining unfiltered tissue. The strainer was rinsed and further ground with DMEM to ensure all single cells were filtered. The flow-through was centrifuged at 450 g for 5 min, and the supernatant was removed. If red blood cells (RBCs) were present in the pellet, 10–20 mL of 1 × MACS RBC lysis buffer (prepared by diluting 10 × MACS RBC lysis buffer [MACS, 130–094–183] in Milli-Q water) was added and incubated at room temperature for 10 min. To stop RBC lysis, 20 mL of DMEM was added, and the mixture was centrifuged at 450 g for 5 min. The supernatant was discarded, and the cell pellet was washed with 10 mL of 4 °C DMEM. After another centrifugation at 450 g for 5 min, the supernatant was discarded, and the cells were resuspended in cold PBS (Sigma, D8537) with 0.04% BSA solution (Ambion, AM2616) and passed through a 40-μm Flowmi cell strainer (Bel-Art, h13680-0040). Total cell counts and viability were assessed using both an automated cell counter (TC20™, Bio-Rad) and a hemocytometer. The cells were washed in PBS with 0.04% BSA and resuspended to a concentration of approximately 1000 cells/μL. Single-cell suspensions were then subjected to droplet-based scRNA-seq (10X Genomics), targeting an average of 10,000 cells per sample.

### Single cell capture and library preparation for scRNA-seq

Single cells were captured in droplet emulsions using the GemCode Single-Cell Instrument (10 × Genomics), following the single cell 3’ manufacturer’s protocol. The machine partitions thousands of cells into nanoliter-scale gel bead-in-emulsions individually, where cDNA libraries were generated and barcoded with a 10X barcode and unique molecular identifier (UMI). The gel bead-in-emulsions were then broken, and the silane magnetic beads were used to clean up the gel bead-in-emulsion reaction mixture. Barcoded, full-length cDNAs were then amplified via PCR, and the libraries were pooled for deep sequencing by Illumina NovaSeq 6000 to achieve 75-bp reads.

### Quality control, clustering, and normalization

Illumina output from 10X Genomic Chromium sequencing reads were initially generated and pre-processed using CellRanger (v.3.1.0, 10 × Genomics). This pre-processing involved demultiplexing cellular barcodes, performing read alignment, and creating a gene count matrix. Subsequently, quality control measures were implemented for each sample, which involved filtering out cells on the basis of criteria such as the number of expressed genes, total UMI counts, and the proportion of total UMI counts originating from mitochondrial genes using the Seurat package [33]. Following quality control, data normalization was performed using the "NormalizeData" function with default settings. To identify the most variable genes, we applied the "FindVariableFeatures" function. To ensure comparability across different scRNA-seq samples within the same study, we executed scale normalization using the "ScaleData" function to harmonize total UMI counts. Integration through the Harmony method was investigated using the “RunHarmony” function with default settings. A principal component analysis and uniform manifold approximation and projection (UMAP) [34] were sequentially carried out using the "RunPCA" and "RunUMAP" functions, respectively. Cells were then grouped into different clusters in the embedding space via the "FindCluster" function.

### Cell type annotation

We applied inferCNV to each scRNA-seq sample to determine gene expression intensity across the genome and identify regions that contained somatic large-scale chromosomal copy number alterations [35]. We identified clusters of cells showing high levels of inferred copy number aberrations, either gains or losses, and classified them as tumor cells. Non-tumor cells were identified through supervised learning [36] using reference scRNA-seq data annotated from a BC study [37]. To facilitate cell type annotation, we initiated the process by identifying anchors between the reference and integrated datasets using the "FindTransferAnchors" function after performing log normalization. We subsequently mapped 2 types of annotations, major with 11 cell types and minor with 33 cell types, to the query data from the reference profile using the "MapQuery" function from the SingleR package. For T cell subtypes, we employed the minor annotation, while for other cell types, such as B cells, NK cells, and myeloid cells, we conducted sub-clustering and subtype identification based on marker gene expression [37].

### Trajectory analysis

We leveraged Monocle 3 [38] (version 1.3.4) for the construction of the pseudo-time trajectory analysis, focusing on tumor cells from both liver and pleural effusion metastatic sites. To ensure the comparability of pseudotime across samples from different patients, we employed Harmony integration to correct batch effects. The clustering of cells was accomplished using the "cluster_cell" function, followed by the fitting of a principal graph within each partition via the "learn_graph" function, all within the UMAP embedding space.

### Differentially expressed gene detection and functional analysis

To identify differentially expressed genes between cells in different comparisons, we utilized the "FindMarkers" function in Seurat, focusing on the most significantly differentially expressed genes, with adjusted *p*-values < 0.01. We conducted a pathway analysis using the fgsea package [39]. For each category of comparison, we selected the top 5 pathways with the highest normalized enrichment score. Specifically, for tumor cells, we explored hallmark and gene ontology biological process pathways. For CD4^+^ and CD8^+^ T cells, we used gene signatures provided by Chu et al. [40]. The M1 and M2 macrophage subtypes were distinguished using signatures from Azizi et al. [41]. The exhaustive T cell signatures were obtained from Tietscher et al. [42].

### Module score analysis

To calculate module scores for specific gene sets, we employed the "addModuleScore" function, which aggregates the normalized gene expression of all the genes in the gene set. The module-specific gene sets represent the important functional gene components of the biological process of interest. For tumor and non-tumor cells analyses, we adopted meta-programs (MP) signatures on the basis of the work of Gavish et al. [43]. These signatures were generated to comprehensively define the functional states and interactions of cancer cells and other cell types, enabling detailed characterization of each cell types of functions, context specificity, and interactions. The median of the difference between paired comparisons (e.g., sample status or metastatic sites) was evaluated.

### Cell communication analysis

A cell communication analysis was performed using the R package CellChat [44]. The "CellchatDB.human" database was set as the ligand-receptor interaction database. Overexpressed signaling genes and ligand-receptor interactions (pairs) were identified using the "identifyOverExpressedGenes" and "identifyOverExpressedInteractions" functions. The communication probability between any interaction cell groups, with a minimum cell number of 10, was calculated using the "computeCommunProb" function. By summarizing all related ligands and receptors, the communication probability at the signaling pathway level was calculated using the "computeCommunProbPathway" function.

### Preparation of patient samples for bulk RNA-seq and data processing

In the validation study of the MD Anderson cohort, total RNA of fresh tissues was extracted with Trizol reagent (Ambion Life Technologies, 15,596,026) following the manufacturer’s instructions. RNA purity and concentration were measured using a NanoDrop 2000c spectrophotometer (Thermo Fisher Scientific). In FFPE samples, RNA-Seq libraries were generated using the TruSeq RNA Exome (Illumina), formerly known as the TruSeq RNA Access Library Prep Kit (Illumina). These libraries were sequenced using the Illumina NextSeq 500 and NovaSeq 6000 at the MD Anderson Advanced Technology Genomics Core. Preparation of RNA libraries and transcriptome sequencing of fresh tissues were conducted by Novogene Corporation, Inc., following standard Illumina protocols.

The tissue and FFPE samples from the MD Anderson cohort were processed separately using the same pipeline. Raw paired-end reads in fastq format were checked for read quality using FastQC/0.11.8 [45]. The Illumina TruSeq adapters were trimmed from the paired-end reads using cutadapt/1.18 [46]. The trimmed reads were then aligned to GENCODE human reference genome GRCh38 using tophat2/2.1.1 [47]. "FeatureCounts" (Subread/1.6.3) was applied to count reads that were mapped to each gene [48]. Genes were annotated using the gene transfer format file for GENCODE reference GRCh38. We filtered out unexpressed or weakly expressed genes by keeping the genes with more than 0.5 read count per million in at least 2 samples, with adjustment for library size. The read counts for all samples were normalized using the trimmed mean of M method implemented in the R Bioconductor package edgeR to generate the abundance for each gene [49]. As recommended by the Cibersortx tutorial, the counts-per-million values were calculated without log2 transformation to be consistent with the scRNA-seq data input. The gene names were converted to HUGO gene symbols, and the gene duplicates with lower expression levels were removed.

### Korean cohort data processing

The second validation cohort comprised published bulk RNA-seq data from a clinical trial (NCT03401359) involving Korean patients with HR+/HER2- mBC [50]. This cohort included patients with recurrent and/or mBC treated with palbociclib plus an aromatase inhibitor or fulvestrant (with a gonadotropin-releasing hormone agonist for premenopausal patients) at Samsung Medical Center and Seoul National University Hospital from 2017 to 2020. Patients who refused to provide informed consent or withdrew from the study were excluded. For the validation study, we used bulk RNA-seq data from 61 BL tumor biopsies obtained prior to CDK4/6i treatment, including 22 fresh tissue biopsies and 39 FFPE samples. In this cohort, 72% of patients received first-line treatment with palbociclib plus an aromatase inhibitor, while the remaining patients received palbociclib plus fulvestrant or other selective estrogen receptor degraders (Table 3). Approximately 59% of biopsies were from breast tissue, with the rest from metastatic tissues, including liver, bone, and lung (Table 3). All patients were of Korean descent, and PFS duration data were obtained from all participants.

**Table 3:** Charateristics of Patients in the Korean Cohort (*n* = 61)

Parameter	N (%)
**Mean age (range), y**	48 (30.7–65.9)
**CDK4/6i course**	
Palbociclib	61 (100%)
Abemaciclib	0
Ribociclib	0
**Endocrine therapy course**	
Aromatase inhibitor	44 (72.1%)
Fulvestrant	6 (9.8%)
Others	11 (18.1%)
**Mean duration of CDK4/6i + ET treatment, month (range), m**	13.2 (0.7–39.8)
**Form of biopsy specimens**	
Fresh tissue	25 (41.0%)
FFPE	36 (59.0%)
**Prior treatment**	
*De novo*	22 (36.1%)
Neo/Adjuvant chemotherapy	39 (63.9%)
**Source of Biopsy**	
Breast	38 (59.0%)
Liver	6 (9.8%)
Bone	4 (6.6%)
Lung	3 (4.9%)
Others	12 (19.7%)

The RNA-seq by Expectation Maximization (RSEM) expected read counts for the Korean cohort dataset were obtained from Dr. Yeon Hee Park. The processing of the count data, including gene filtering, data normalization, transformation, and gene name conversion, was the same as described above to ensure consistency in data handling.

### Cibersortx cell type deconvolution analysis

The scRNA-seq data, including the count matrix with cell barcode and feature information and cell metadata, were downloaded from GSE176078 [37]. The data were subsetted to include only HR+ samples, excluding the normal epithelial category and other unrelated cell types. This made the scRNA-seq data comparable with our bulk RNA-seq data. Consistently, HUGO gene symbols were obtained, and gene duplicates with lower expression levels were removed to maintain consistency in gene annotation. The processed scRNA-seq data were supplied as input to Cibersortx (https://cibersortx.stanford.edu/) to generate a signature matrix. Using the generated scRNA-seq signature matrix and the bulk RNA-seq data as the mixture file, Cibersortx was run in the relative fraction mode to impute cell fractions. S-mode batch correction was applied to correct for differences between the scRNA-seq and bulk RNA-seq platforms. Next, we imputed tumor epithelial cell type gene expression using the high-resolution mode in Cibersortx. Target signature genes of interest were uploaded for expression imputation. S-mode batch correction was enabled in this step to ensure accurate imputation across different data platforms.

### Gene signature score calculation and signature validation

A bar plot was created to display the distributions of cell type proportions between samples, utilizing the relative fractions output from Cibersortx. Next, for the imputed gene expression data for cancer epithelial cell type, genes with missing values or values equal to 1 in all samples were removed to exclude those that were not expressed or lacked sufficient statistical power for imputation. The remaining gene expression values were log2-transformed and standardized. For the combined RNA-seq data, batch effects (tissue or FFPE) were removed using the "removeBatchEffect" function from limma package [51] before standardization.

Average scores for each patient were calculated for BL tumor cells up signature genes. Not all signature genes were found in the imputed gene expression data; thus, only the available genes were used for score calculation. Patients were divided into gene signature–high and –low score groups using the median gene signature scores as the cutoffs. The association between scores (high or low) and PFS duration was evaluated at 2- or 3-years using Kaplan–Meier (KM) survival curves and the log-rank test.

### Integration of bulk RNA-seq data for longitudinal analysis

To validate the increase in CD8^+^ T and NK cells observed in responder patients from the scRNA-seq analysis, we analyzed a publicly available bulk RNA-seq dataset from the Korean cohort [50]. We selected 2 patients with longitudinally matched breast tumor samples collected before and after progression on CDK4/6is. One patient was categorized as an LP with a PFS of 10.1 months, while the other was an EP with a PFS of 2.8 months. Cell type deconvolution analysis was performed using Cibersortx as previously described. An alluvial analysis was conducted using ggplot2/3.5.1 [52] to visualize longitudinal changes in immune major populations.

### Statistical analyses

GraphPad Prism (version 10), IBM SPSS Statistics (version 29.0.0), and R (version 4.2.0) software packages were used to analyze the data. Statistical analyses were performed using the student's t-test for two-group comparisons and one-way ANOVA with the Dunnett test for multiple group comparisons (*, *p* < 0.05; **, *p* < 0.01; ***, *p* < 0.001; ****, *p* < 0.0001).

## Results

### Transcriptomic landscape of the TME in patients with HR+/HER2- mBC before and after CDK4/6i treatment

To investigate the intratumor microenvironment of patients with HR+/HER2- mBC undergoing CDK4/6i treatment, we collected biopsies from metastatic sites at before CDK4/6i therapy (baseline, BL, *n* = 8) or at the onset of disease progression (*n* = 10) for the scRNA-seq study. The BL samples were collected from patients who responded favorably to CDK4/6i treatment, with a median progression-free survival (mPFS) duration of 25.5 months. Progression biopsies were categorized as early progressors (EP, mPFS = 3.0 months; *n* = 3) or late progressors (LP, mPFS = 11.0 months; *n* = 7) using a PFS cutoff of 6 months (Fig. 1A). This threshold was chosen based on its use in prior studies [16, 26], both of which established the utility of this classification for identifying distinct molecular and clinical phenotypes of resistance. scRNA-seq was performed on all collected samples following stringent quality control measures. Most patients received palbociclib (*n* = 17 [94%]) or abemaciclib (*n* = 1 [6%]) combined with endocrine therapy (Table 1). Biopsies were obtained from various metastatic sites, including the liver (*n* = 9 [50%]), pleural effusion (*n* = 6 [33%]), ascites (*n* = 2 [11%]), and pelvic bone (*n* = 1 [6%]).

After quality control, 83,222 cells were available for analysis, each expressing an average of 1,893 genes and 8,549 unique molecular identifiers (UMIs). As shown in the study workflow (Fig. 1B), we analyzed tumor and non-tumor cells separately and stratified samples based on metastasis sites for downstream analyses, including clustering, cell type annotation, differential expression gene analysis, and ligand-receptor interaction analysis.

To distinguish tumor from non-tumor cells, we utilized InferCNV [53], which infers copy number variants from scRNA-seq data by identifying regions of the genome that are gained or lost in the tumor cell population (Figure S1A). The canonical markers *EPCAM* and *KRT19* identified tumor cells, while *CD3D*, *CD2*, *PTPRC*, *CD14*, *AIF1*, *CD79A*, and *COL1A2* validated non-tumor cells. UMAP was used to reduce dimensionality and categorize cells with similar expression patterns (Figs. 1C and S1B). The proportion of tumor cells in each sample is shown in Fig. 1D. Both UMAP and Harmony integration plots [54] confirmed the segregation of tumor (*n* = 36,659) and non-tumor (*n* = 46,563) cells (Fig. 1E). The distribution of cells across the BL, EP, and LP groups was delineated, with 38,493 in the BL, 13,536 in the EP, and 31,193 in the LP groups (Fig. 1F).

A differential gene expression analysis highlighted distinct profiles among study groups (Fig. 1G and Table S1). BL tumor cells exhibited elevated expression of *MGP* (matrix Gla protein), *AZGP1* (alpha-2-glycoprotein 1), *AGR2* (anterior gradient protein 2), *TFF3* (trefoil factor 3), and *FGFR1* compared to EP and LP cells (Figs. 1G and S1C,D). The top differentially expressed genes in BL cells formed a predictive biomarker panel for CDK4/6i treatment outcomes in HR+/HER2- mBC (Fig. 1H), which was subsequently utilized in validation studies. EP cells displayed upregulation of *LY6E* (lymphocyte antigen 6E), *S100A9* (S100 calcium binding protein A9), *LGALS3* (galectin 3), and *CLU* (clusterin) genes relative to BL cells (Figure S1C). LP cells showed increased expression of *TIMP1*, a metallopeptidase-dependent regulator of growth and apoptosis, and *VIM*, implicating their role in epithelial-mesenchymal transition (EMT) and tumorigenesis (Figures S1D and S1E).

A gene set enrichment analysis (GSEA) revealed key pathways involved in tumor progression. Both EP and LP cells had reduced estrogen response pathways compared to BL. LP samples exhibited increased PI3K, Myc, IFN-γ, and complement pathway activities relative to BL samples (Fig. 1I). LP cells also showed increased inflammatory response, KRAS, mitotic, and TNF-α pathway activities compared to EP cells, suggesting different mechanisms in early versus late progression under CDK4/6i treatment. A complementary meta-program (MP) analysis supported these functional insights (Figure S1F) [43]. These findings highlight distinct gene expression profiles that vary with CDK4/6i treatment stage and disease progression in the TME of patients with HR+/HER2- mBC.

### Identification of metastatic site-specific gene expression patterns in tumor cells

**Fig. 2:**
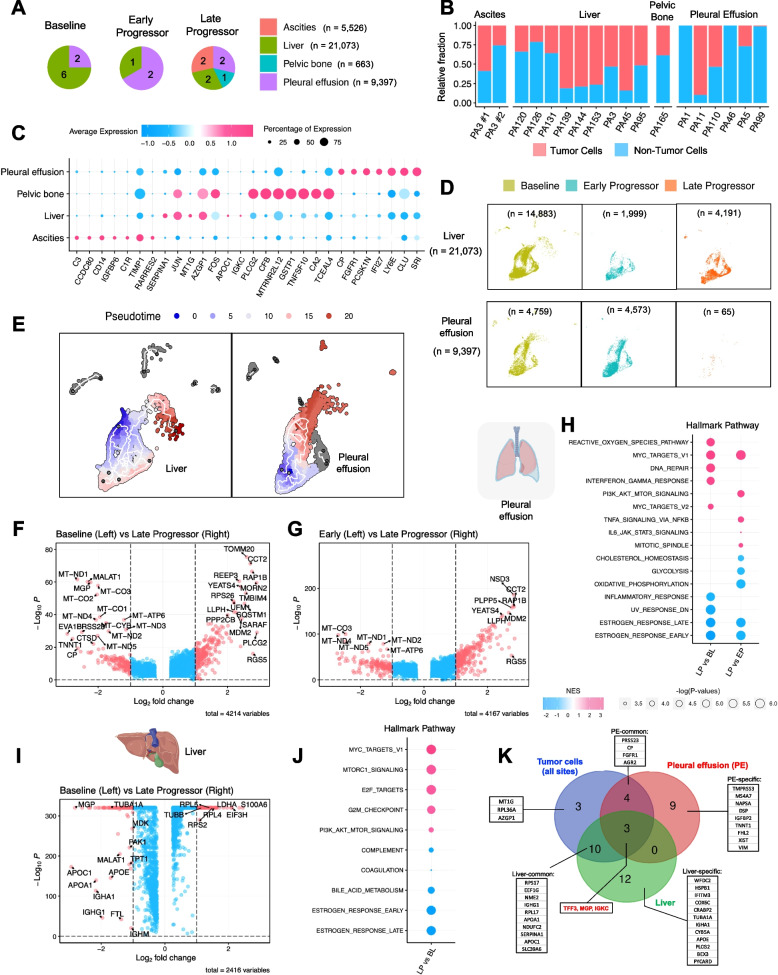
Tumor cells stratified by metastatic site. **A** Pie chart displaying the frequency of metastatic sites, stratified by different sample statuses (BL, EP, and LP). **B** Bar plot showing the relative fraction of tumor versus non-tumor cells for each sample, stratified by different metastatic sites (ascites, liver, pelvic bone, and pleural effusion). **C** Dot plot depicting the top 7 differentially expressed genes across various metastatic sites. **D** UMAP plot of tumor cells from the liver and pleural effusion, stratified by different sample statuses (BL, EP, and LP). **E** Trajectory analysis of liver and pleural effusion samples using Monocle 3. **F** Volcano plot showing differentially expressed genes between BL (left) and LP (right) samples with pleural effusion metastasis. The x-axis indicates the log_2_ fold changes, while the y-axis represents the negative log P values. A positive log_2_ fold change indicates genes are upregulated in the LP samples. **G** Volcano plot showing differentially expressed genes between the EP (left) and LP (right) samples with pleural effusion metastasis. **H** Hallmark GSEA showing the normalized enrichment score from pleural effusion samples. **I** Volcano plot showing differentially expressed genes between the BL (left) and LP (right) samples from the liver metastases. **J** Hallmark pathway analysis showing the normalized enrichment score from samples with liver metastases. **K** Venn diagram showing overlapping and unique genes from pleural effusion or liver metastases in BL samples

Given the differences in the TME across metastatic sites, we hypothesized that gene expression patterns in tumor cells vary by site. We categorized samples by metastasis site: liver, pleural effusion, ascites, and pelvic bone (Fig. 2A). In the BL group, 2 biopsies originated from pleural effusion (29%), and 6 were from the liver (71%). In the EP group, 1 sample was collected from the liver (33%), and 2 were from pleural effusion (67%). In the LP group, 2 samples were from pleural effusion (29%), 2 from ascites (29%), 2 from the liver (29%), and 1 from the pelvic bone (13%) (Fig. 2A). The distribution of tumor and non-tumor cells by metastatic site is shown in Fig. 2B. A heatmap of the top 15 upregulated genes in each group BL, EP and LP indicates tissue-specific tumor signatures (Figure S2A). While the genes in BL and EP are broadly overexpressed across tumor cells, those in LP are upregulated in only a small subset of cells from ascites but not in pelvic bone. This observation suggests intratumor heterogeneity and reflects the influence of diverse metastatic sites on gene expression patterns. These results underscore the tissue-specific nature of gene expression and highlight the importance of considering metastatic microenvironments in resistance mechanisms. Metastatic site-specific biomarkers are presented in Fig. 2C. To mitigate tissue-dependent factors, samples from the same site were stratified by BL, EP, and LP (Fig. 2D), and differential gene expression was analyzed.

A trajectory analysis of liver and pleural effusion metastasis showed tumor cells' progression from BL to EP and LP stages along pseudotime (Fig. 2E). For pleural effusion, BL and EP tumor cells had higher expression of mitochondria-associated genes, such as *CO* (cytochrome C oxidase), *ND* (NADH dehydrogenase), and *ATP6* (Fig. 2F and Table S2), while LP cells expressed EMT-associated genes, such as *YEATS4*, *MDM2*, and *RGS5* (Fig. 2G and Table S2). A GSEA hallmarks analysis revealed that LP cells, compared to BL or EP, had downregulated estrogen response pathways but upregulated Myc, reactive oxygen species, DNA repair, and IFN-γ pathways. LP cells also showed enrichment for the mitotic, PI3K, and TNF-α pathways (Fig. 2H).

BL liver metastases significantly differed from pleural effusion. Unlike the mitochondria-associated genes in pleural effusion, BL liver metastases had elevated apolipoprotein genes, such as *APOC1*, *APOA1*, and *APOE* (Figs. 2I and S2B and Table S2). LP liver metastasis cells had higher expression of genes linked to extracellular exosomes promoting EMT and metastasis, such as *S100A8*, *EIF3H*, *LDHA*, *PPA1*, and *ENO1*. They also showed upregulation of RPL/RPS family genes (previously identified in breast tumor metastases to the brain [55]) and proliferation-associated genes, such as *TUBB*, compared to BL (Figs. 2I and S2C and Table S2). Consistent with the pleural effusion findings, a GSEA hallmark analysis showed decreased estrogen response but increased Myc, mTOR, E2F, PI3K, and G2/M checkpoint pathways in LP compared to in BL liver metastases (Fig. 2J).

While metastatic site-specific profiles were identified among the BL, EP, and LP samples (Figures S2D and S2E), we also found common biomarkers in BL tumor cells, irrespective of their metastatic site. These markers, including *TFF3* and *MGP*, are potential to predict treatment outcomes independent of tissue resources (Figs. 2K and 1H). For tissue-specific biomarkers, *TMPRSS3* (transmembrane serine protease 3), *NAPSA* (napsin A aspartic peptidase), and *DSP* (desmoplakin) were identified from pleural effusion BL tumor cells, while *APOE* (apolipoprotein E), *COX6C* (cytochrome C oxidase subunit 6C), and *WFDC2* (WAP 4-disulfide core domain 2) were identified from liver BL tumor cells (Fig. 2K). These results highlight distinct metastatic site-specific gene expression patterns in HR+/HER2- mBC, identifying unique biomarkers that could potentially predict CDK4/6i treatment outcomes and emphasize the need for site-specific therapeutic strategies.

### Non-tumor cell atlas in the microenvironment of HR+ mBC before or after treatment with CDK4/6is

**Fig. 3:**
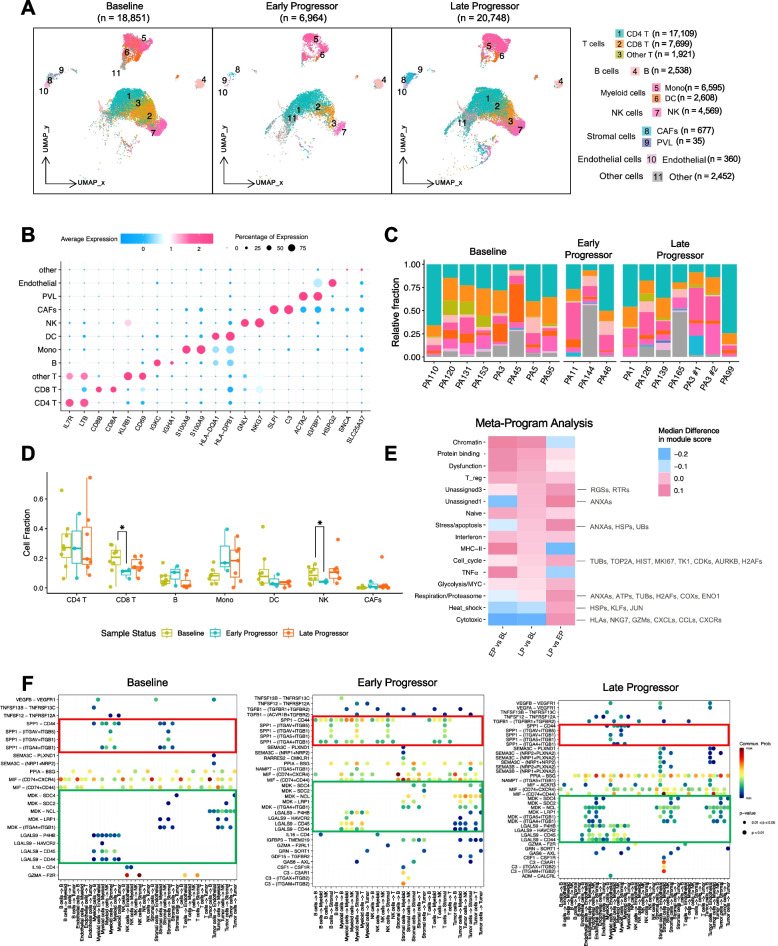
Analysis of major non-tumor cell subtypes. **A** UMAP plot of major non-tumor cells stratified by different sample statuses (BL, EP, and LP). **B** Dot plot showing the marker gene expression for each major non-tumor cell type in (A). **C** Bar plot showing the relative fractions of major non-tumor cell types for each sample. The color legend is the same as in panel (A). **D** Cell fraction frequency of major non-tumor cell types across sample statuses (BL, EP, and LP). **E** MP analysis heatmap showing the median module score difference from samples with pooled T cells. **F** Ligand-receptor (L-R) interaction analysis using CellChat in samples from BL, EP, and LP. The red box indicates the SPP1-CD44 or ITGA4 interactions, while the green box indicates the MDK-NCL, MDK-syndecan, or LGALS9 interactions. *, *p* < 0.05

Recognizing the significant influence of non-tumor cells in the TME on CDK4/6i response [56], we examined the composition and transcriptomic profiles in HR+/HER2- BC metastases before and after CDK4/6i treatment. Using supervised and marker-based approaches [37], we annotated specific cell subpopulations: CD4^+^ T cells (*n* = 17,109), CD8^+^ T cells (*n* = 7,699), B cells (*n* = 2,538), monocytes (*n* = 6,595), dendritic cells (DCs) (*n* = 2,608), natural killer (NK) cells (*n* = 4,569), cancer-associated fibroblasts (*n* = 677), perivascular-like cells (*n* = 35), endothelial cells (*n* = 360), and other cell types (*n* = 2,452) (Fig. 3A and Table S3). Dot plots in Fig. 3B show scaled expression levels and proportions of cells with cluster-specific markers, while Fig. 3C depicts cell type composition across each sample.

Notably, CD8^+^ T cell frequencies were significantly higher in BL and LP samples than in EP samples, suggesting that CD8^+^ T cell frequency is a potential biomarker for predicting CDK4/6i response (Fig. 3D). The lowest CD8^+^ T cell frequency in EP indicates less responsiveness to CDK4/6i treatment. NK cells showed a similar pattern, with significant higher frequencies in BL and LP than in EP cells (Fig. 3D). Monocyte frequencies were increased in EP and LP samples compared to in BL samples, and the DC population varied across the EP, LP, and BL samples (Fig. 3D).

The MP analysis of T cells indicated upregulated chromatin and protein binding and T cell dysfunction in EP and LP samples compared to in BL samples, but downregulated heat shock protein expression and T cell cytotoxicity (Fig. 3E). These findings suggest that progressing tumors are associated with T cell dysfunction and decreased cytotoxicity. Compared to EP, LP T cells showed upregulated heat shock protein expression and cytotoxicity but downregulated TNF-α and MHC-II, indicating distinct T cell-mediated mechanisms between EP and LP with CDK4/6is (Fig. 3E).

Given the increased binding and interactions on progressor T cells, we investigated ligand-receptor (L-R) interactions using CellChat [44]. The data revealed enhanced osteopontin (SPP1-CD44) and SPP1- (ITGA4 + ITGB1) interactions between T and NK cells in EP samples compared to in BL and LP samples (Figs. 3F and S3A-C). The SPP1-CD44 interaction is known to impede sustained T cell proliferation [57] and promote tumor progression in various cancers, including glioma [58], gastric cancer [59], prostate cancer [60], and pancreatic cancer [61]. Additionally, L-R interactions involving midkine (MDK)-nucleolin (NCL), MDK-syndecan, and galectin 9 (LGALS9)-CD44 were enhanced in LP. MDK-NCL induces an immunosuppressive microenvironment [62], while LGALS9-CD44 enhances regulatory T cell stability and function [63]. These findings highlight the differential expression patterns and cellular communication mechanisms that contribute to an immunosuppressive microenvironment in progressors, with potential implications for overcoming tumor progression during CDK4/6i treatment.

### Increased CD8^+^ TILs with elevated cell stress and exhaustion in BL responder patients

**Fig. 4:**
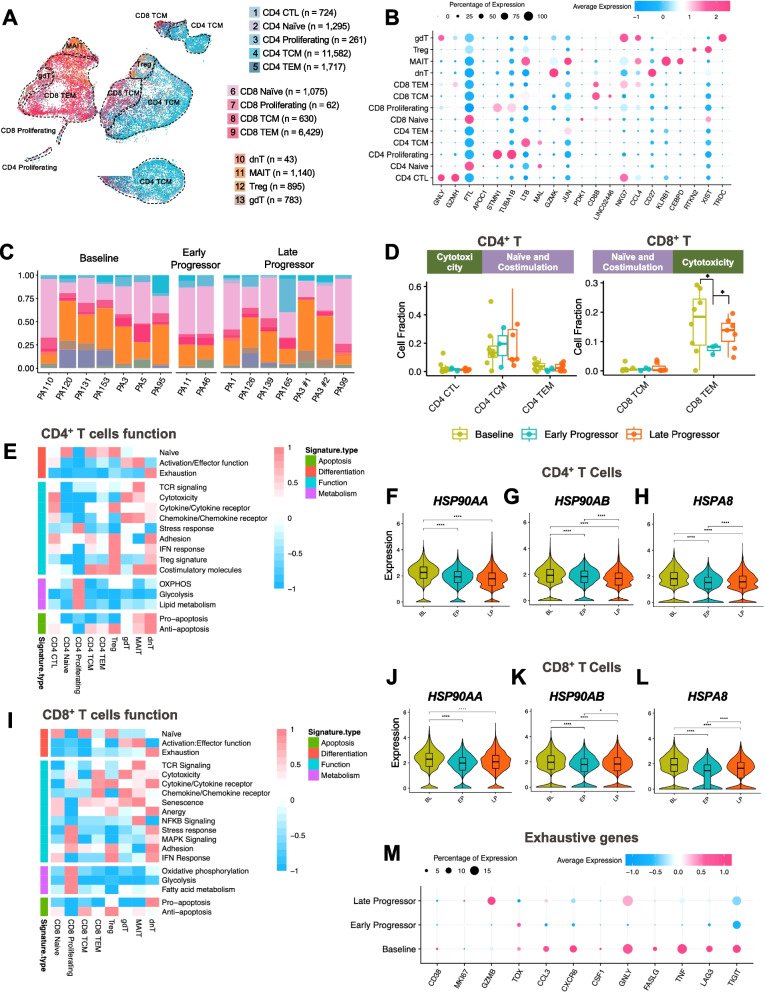
CD4^+^ T and CD8^+^ T cell functional analysis. **A** t-Distributed stochastic neighbor embedding (t-SNE) plot of 13 different subtypes of T cells in the embedding space. **B** Dot plot showing the marker gene expression of each T cell subtype in (**A**). **C** Bar plot showing the relative fraction of each T cell subtype for each sample, stratified by different sample statuses (BL, EP, and LP). **D** Cell fraction of CD4^+^ and CD8^+^ T cell subtypes in BL, EP, and LP samples. **E** Heatmap displaying expression of 14 curated gene signatures across CD4^+^ T cell clusters. Violin plots show significantly differentially expressed stress genes of (**F**) *HSP90AA*, (**G**) *HSP90AB*, and (**H**) *HSPA8* across different groups for CD4^+^ T cells. **I** Heatmap displaying expression of 14 curated gene signatures across CD8^+^ T cell clusters. Violin plots showing that (**J**) *HSP90AA*, (**K**) *HSP90AB*, and (**L**) *HSPA8* were significantly differentially expressed across different groups for CD8.^+^ T cells. **M** Dot plot showing the expression of exhaustive gene signatures across different groups. *, *p* < 0.05; **, *p* < 0.01; ***, *p* < 0.001; ****, *p* < 0.0001

To investigate T cell subtypes in the TME and their gene expression differences before and after CDK4/6i treatment, we classified T cells into various subtypes using established BC annotations [37]. Figures 4A and S3D show the 5 CD4^+^ and 4 CD8^+^ subtypes, with marker genes provided in Figs. 4B, S3E and Table S3. The cell numbers for each cell type were shown in Figure S3F. The proportion of immune cells across BL, EP, and LP stages was shown in Fig. 4C.

A quantitative analysis revealed a trend of increasing central memory CD4^+^ T (T_CM_) cells after treatment (Fig. 4D). CD8^+^ effector memory (T_EM_) cells were significantly increased in BL and LP samples, suggesting that they are predictive of favorable responses to CDK4/6i treatment (Fig. 4D). Higher levels of gamma delta T (gdT) and mucosa-associated invariant T (MAIT) cells were seen in BL and LP tumors compared to EP tumors (Figure S3G).

A functional analysis using established T cell functional gene signatures related to apoptosis, differentiation, and metabolism [40] validated the CD4^+^ and CD8^+^ cell subtype annotations (Fig. 4E). CD4^+^-proliferating cells showed increased expression of genes associated with stress response, MAPK signaling, oxidative phosphorylation, glycolysis, and fatty acid metabolism. Recent studies show that T cells expressing stress-related genes, such as heat shock proteins, are associated with a poor response to immune checkpoint inhibitors, particularly anti-PD-1/PD-L1 therapy, and can be utilized as predictors for lack of response to immunotherapy [40]. Notably, CD4^+^ T cells from BL samples had higher stress/apoptosis gene scores than did LP cells (Figures S4A and S4B). The heat shock genes *HSP90AA1*, *HSP90AB1*, and *HSPA8* were significantly increased in BL compared to EP and LP cells (Figs. 4F-H), suggesting increased cellular stress and replication in BL cells (i.e. prior to CDK4/6i treatment) and indicating more favorable outcomes with immunotherapy after CDK4/6i treatment.

Similar assessments in CD8^+^ T cells showed that proliferating CD8^+^ T cells also expressed genes associated with stress and metabolic processes (Fig. 4I). Despite the high CD8^+^ T cell frequency in BL cells (Fig. 4D), these cells exhibited increased stress gene upregulation, including *HSP90AA1*, *HSP90AB1*, and *HSPA8* (Figs. 4J-L), suggesting resistance to anti-PD-1/PD-L1 therapy compared to tumors that progressed on CDK4/6i. Additionally, the CD8^+^ T cells from BL samples had higher stress/apoptosis gene scores compared to LP cells (Figures S4C and S4D). Double-negative T (dnT) cells also expressed stress, anergy, and exhaustion genes (Fig. 4I). Significantly higher levels of exhaustion genes [42], including lymphocyte-activation gene 3 (*LAG3*), granulysin (*GNLY*), T cell immunoreceptor with Ig and ITIM domains (*TIGIT*), and *TNF* in T cells were found in BL than in EP and LP cells (Fig. 4M), indicating the potential benefit of blockade of novel immune checkpoint receptors LAG3 and TIGIT for BL patients.

To further investigate the composition of T cells in different metastatic sites, we stratified different types of T cells into ascites, liver, pelvic bone, and pleural effusion groups (Figure S4E). The T cell type composition, based on metastatic sites, is shown in Figure S4F. In liver metastasis BL T cells, granzyme family genes (*GZMA*, *GZMH*, and *GNLY*) were enriched, while pleural effusion BL T cells showed increased *TFF3*, *AGR2*, *MGP*, and *MIF* expression (Figure S4G). LP T cells in liver metastasis had increased expressions of *JUN* family genes, while ribosomal genes (*RPL* and *RPS*) expressions were higher in pleural effusion LP T cells (Figure S4H). These findings underscore the critical role of cellular stress response and replication in predicting and enhancing the efficacy of immunotherapy in patients treated with CDK4/6is.

### Transcriptional profiles of NK, B, and myeloid cells

**Fig. 5:**
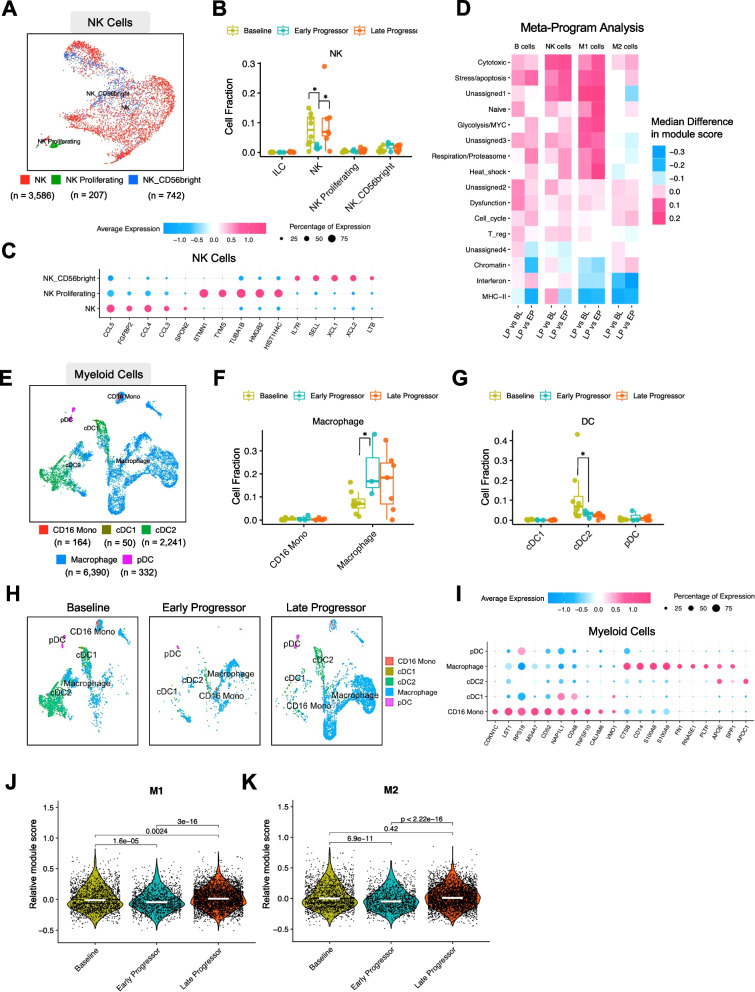
Other non-tumor cells atlas. **A** UMAP plot of NK cells stratified by 3 different NK subtypes. **B** Comparison of NK cell subtype fractions among BL, EP, and LP samples. **C** Dot plot showing the marker gene expression of each NK cell subtype in (**A**). **D** Heatmaps of module score differences from samples with NK cells, M1 macrophages, M2 macrophages, and B cells. (**E**) UMAP plot of myeloid cells stratified by 5 different cell subtypes. **F** Comparison of macrophage cell subtype fractions among BL, EP, and LP samples. **G** Comparison of DC subtype fractions among BL, EP, and LP samples. **H** UMAP plot of annotated myeloid cells stratified by BL, EP, and LP samples. **I** Dot plot showing the marker gene expression for each myeloid cell subtype in (**D**). **J** Module score analysis of M1 macrophages among BL, EP, and LP samples. **K** Module score analysis of M2 macrophages between BL, EP, and LP samples. *, *p* < 0.05

To investigate the roles of different immune cell types in the TME of HR+/HER2 mBC tissues before and after CDK4/6i treatment, we utilized established biomarkers to annotate NK cells, macrophages, and DCs (Table S3). The UMAP plots that depict 3 subtypes of NK cells are shown in Fig. 5A. We observed a significant decrease in NK cells in EP samples compared to in BL and LP samples (Fig. 5B), similar to the trend observed for CD8^+^ cytotoxic T cells (Fig. 4D) and suggesting a potential mechanism for early progression on CDK4/6is, linked to reduced populations of cytotoxic NK and CD8^+^ T cells. Therefore, NK cells could also serve as predictors for late progression. Biomarker genes for each NK cell subtype are depicted in Fig. 5C. While NK cell counts were increased in BL and LP samples compared to in EP, an MP analysis showed that LP NK cells exhibited higher cytotoxicity and stress/apoptosis levels than did BL NK cells (Fig. 5D).

For myeloid cells, including DCs and macrophages, the UMAP plots depicting the 5 subtypes are illustrated in Fig. 5E and Table S3. Following CDK4/6i treatment, the macrophage numbers significantly increased (Fig. 5F), whereas cDC2 cells decreased (Fig. 5G). The UMAPs of myeloid cells showed the expansion of the macrophage population in blue and the decreased cDC2 population in green in LP, as illustrated in Fig. 5H. Biomarker genes for each myeloid subtype are presented in Fig. 5I. Given that cDC2 cells are known for priming naïve CD4^+^ T cells through antigen presentation on MHC class II [64], these results suggest inhibited antigen presentation following CDK4/6i treatment, consistent with the results of the MP analysis (Fig. 5D).

Previous studies have shown that M1 macrophages suppress tumor growth due to phagocytosis and enhanced antitumor inflammatory reactions, whereas M2 macrophages promote tumors through immunosuppression and angiogenesis [65]. To compare the 2 subtypes of macrophages before and after CDK4/6i treatment, we performed a module cores analysis of M1 and M2 macrophage subtypes among BL, EP, and LP samples. Although the M2 scores did not differ between BL and LP cells, LP cells exhibited upregulation in both scores compared to EP cells (Figs. 5J-K). The M1 and M2 subtype identification was shown in Figures S5A-C.

Lastly, we examined B cell populations before and after CDK4/6i treatment, dividing them into naïve, memory, intermediate, and plasmablast subtypes (Figure S5D and Table S3). Quantification of different B cell subtypes revealed that memory and naïve B cell frequencies were decreased in LP samples compared to in BL samples (Figure S5E), suggesting a shift in the immune landscape as the disease progresses.

Taken together, our data indicate that BL responders and LP tumors have higher levels of CD8^+^ cytotoxic T cells and NK cells than EP tumors, implying that these cells are potential predictive biomarkers for CDK4/6i response. Despite higher cytotoxic TILs in BL tumors, we found that stress, apoptosis, and exhaustion genes were upregulated, suggesting a lack of response to immunotherapy. Moreover, the increase in M1 and M2 macrophages and the decrease in memory B and cDC2 cells in LP tumors may be associated with mechanisms of disease progression during CDK4/6i treatment.

### Temporal changes in the intratumor microenvironment from longitudinal biopsies before and after CDK4/6i treatment for HR+/HER2- mBC

**Fig. 6:**
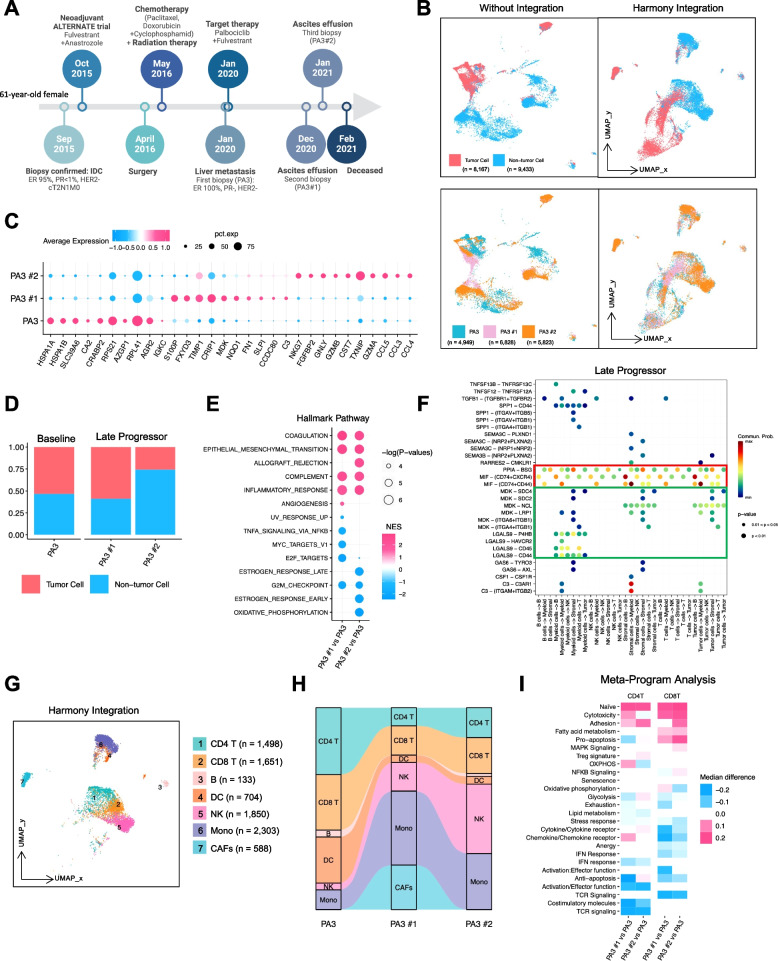
Longitudinal biopsy analysis. **A** Workflow outlining the history of BC diagnosis and collection of longitudinal samples from patient PA3. **B** UMAP plot displaying tumor cells (pink) versus non-tumor cells (blue) with (right) or without (left) Harmony integration. Samples were stratified based on 3 longitudinal biopsies (PA3, PA3#1, and PA3#2). **C** Dot plot showing the top 10 differentially expressed genes across 3 longitudinal biopsies. **D** Bar plot depicting the relative fraction of tumor cells versus non-tumor cells for each sample. **E** Hallmark pathway analysis conducted in tumor cells from each sample. **F** Ligand-receptor (L-R) interaction analysis using CellChat in progression samples. The red box indicates the MIF and CD74 interactions, while the green box indicates the MDK-NCL and LGALS9-CD44 interactions. **G** UMAP plot of major non-tumor cell subtypes with Harmony integration. **H** Alluvial plot demonstrating the dynamic changes in major non-tumor cell subtype fractions across 3 longitudinal samples. The color legend is the same as in panel (**G**). **I** Heatmap from MP module score analysis displaying the median module score difference from pooled CD4^+^ and CD8^+^ T cells, respectively

To examine dynamic changes in the intratumoral microenvironment, we conducted a longitudinal study in 1 patient, a 61-year-old White female, who underwent sequential biopsies before and after CDK4/6i treatment. In September 2015, the patient was first diagnosed with stage IIb (cT2N1M0) ER+/HER2- BC and underwent neoadjuvant therapy with fulvestrant plus anastrozole in a clinical trial, followed by surgery, adjuvant chemotherapy, radiation therapy, and anastrozole as maintenance treatment. She was diagnosed with mBC in June of 2020 and a liver biopsy (PA3) was obtained before initiating her on palbociclib. After 11 months of therapy of palbociclib plus fulvestrant, the patient developed ascites, prompting the collection of 2 subsequent biopsies (PA3#1 and PA3#2, taken 3 weeks apart) from the ascites fluid (Fig. 6A), confirming disease progression.

The patient’s metastatic tumor and non-tumor cells (from the initial liver biopsy and subsequent ascites samples) were separated using InferCNV and validated with canonical markers, as shown in Fig. 1E. The UMAP plot illustrates a clear distinction between the populations of tumor (*n* = 8,167) and non-tumor cells (*n* = 9,433) (Fig. 6B). Stratified UMAP plots for the 3 biopsies showed distinct clusters, with differential gene expression in tumor and non-tumor cells (Fig. 6B). The top 10 differentially expressed genes from each group are presented in Fig. 6C, with the composition of tumor and non-tumor cells depicted in Fig. 6D.

A GSEA Hallmark analysis of the tumor cells revealed that, compared to BL, progressing tumor cells exhibited increased expression of genes associated with EMT, inflammatory response, and complement pathways (Fig. 6E). Conversely, E2F targets and G2/M checkpoint pathways were downregulated in the progressive stages of palbociclib treatment (Fig. 6E). These findings were corroborated by MP analysis, which showed upregulated EMT, interferon pathways, and senescence compared to BL (Figure S6A). Additionally, the initial liver biopsy at the onset of disease progression showed increased epithelial senescence, which decreased in subsequent biopsies. Stress-associated genes were also highly expressed before treatment but not after (Figure S6A).

A ligand-receptor (L-R) interaction analysis revealed enhanced interactions of macrophage migration inhibitory factor (MIF) with CD74/CXCR4 in LP cells compared to in BL samples (Figs. 6F and S6B). Interactions involving MDK with NCL, MDK with syndecan, and LGALS9 with CD44 were also enhanced in LP cells (Figs. 6F, S6C, and S6D), suggesting an immunosuppressive microenvironment that enhances adaptive regulatory T cell function. These findings align with our previous observations (Figs. 3, 4 and 5) of an immune-suppressive milieu in LP cells, indicating potential targets to overcome tumor progression during CDK4/6i treatment.

Next, we identified different subtypes of immune cells based on established biomarkers (Figs. 6G and S6E). There were significantly expanded proportions of NK cells and monocytes/macrophages in biopsies taken after disease progression (Figs. 6H and S6F). Cancer-associated fibroblasts also increased during progression, suggesting their role in mediating palbociclib resistance. Moreover, an MP analysis of CD4^+^ and CD8^+^ T cells showed increased cytotoxic function in progressive biopsies compared to in pre-treatment biopsies (Fig. 6I), consistent with our earlier findings. Notably, heat shock and exhaustion gene expression were downregulated in progression biopsies, implying a potentially favorable response to immunotherapy (Fig. 6I).

Collectively, the results of our longitudinal study revealed dynamic changes in the intratumoral microenvironment with CDK4/6i exposure. These findings underscore the evolution of immune responses, cellular interactions, and gene expression profiles associated with treatment progression, offering insights into potential targets for therapeutic intervention that require validation using independent patient cohorts.

### Validation of scRNA-seq-derived biomarkers in two independent cohorts

**Fig. 7:**
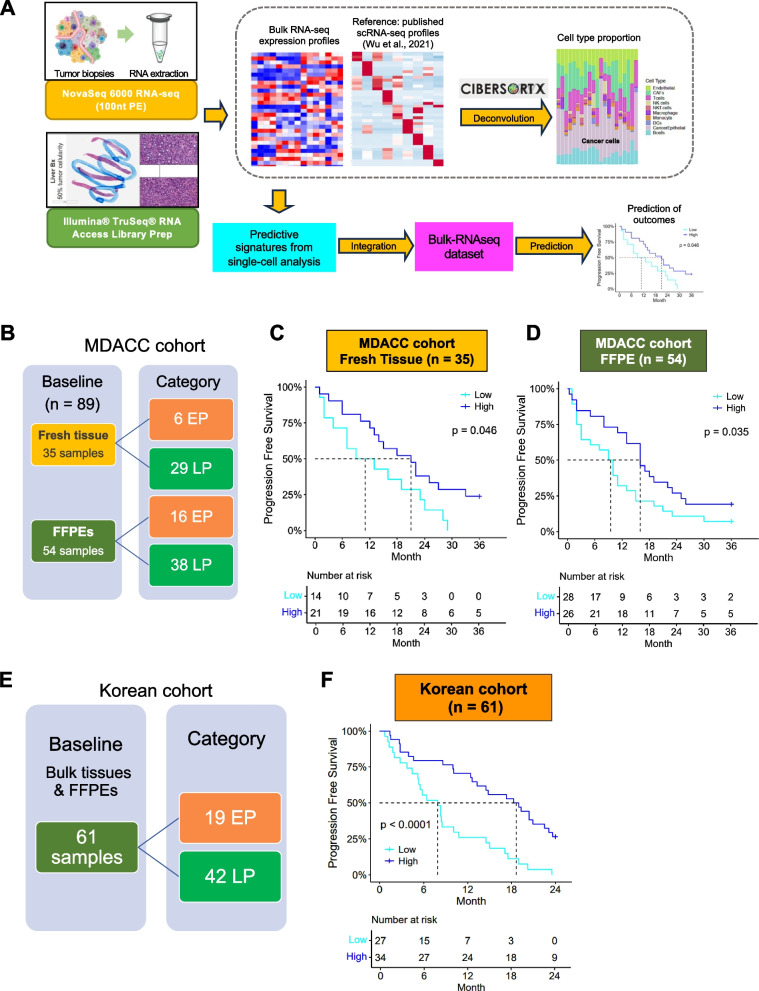
Predictive biomarker signature validation. **A** Workflow detailing the collection process for fresh biopsies and FFPE samples for bulk RNA sequencing and subsequent data processing. **B** Composition of samples in the MD Anderson cohort (*n* = 89). The cutoff for early versus late progression was 6 months. **C** KM plots illustrating mPFS time in the signature-high group compared to the -low group were generated using fresh biopsy RNA-seq data from the MD Anderson cohort (*n* = 35) and (**D**) FFPE RNA-seq data from the MD Anderson cohort (*n* = 54). KM plots, shown with the mPFS duration, were generated utilizing all 13 upregulated gene signatures for tissue samples and 10 upregulated gene signatures for FFPE samples, respectively. **E** Composition of samples in the Korean cohort (*n* = 61). **F** Utilizing bulk RNA-seq data from the Korean cohort (*n* = 61), we generated KM plots with the indicated mPFS durations utilizing 8 upregulated gene signatures. Log-rank test p-values are displayed

All potential biomarkers identified require validation in larger and independent cohorts to assess their predictive value and utility in guiding treatment decisions. Our scRNA-seq analysis identified a panel of biomarkers from BL tumor cells that we hypothesized were predictive of CDK4/6i treatment response (Fig. 1H). This panel comprises 13 genes that were significantly upregulated in the BL samples compared to in the progression samples. To test this hypothesis, we determined whether this gene panel could effectively predict clinical outcomes using bulk RNA-seq data from 2 independent patient cohorts. Figure 7A shows the workflow of using the gene signature from scRNA-seq to predict response in the 2 validation cohorts by bulk RNA-seq.

Briefly, deconvolution of the bulk RNA-seq data was performed using Cibersortx to estimate cell type proportions and imputed gene expression profiles. By focusing on the 13 upregulated genes listed in Fig. 1H, we analyzed the imputed gene expression data specifically in the cancer epithelial cell type to minimize inter-sample TME variability and calculated average scores for each sample. Patients were divided into high and low gene signature score groups using the median value as the cutoff. The association between gene signature scores and median progression-free survival (mPFS) duration was then evaluated.

The first cohort is a retrospective analysis from UT MD Anderson Cancer Center, where we collected 89 BL samples from patients with HR+/HER2- mBC. To determine whether the tissue source (FFPE or fresh biopsies) affected the validation results, we divided the MD Anderson cohort into FFPE and fresh tissue subgroups for evaluation (Fig. 7B and Table 2). In the 35 fresh tissue BL samples, all 13 genes listed in Fig. 1H were imputed. The fresh biopsies showed longer mPFS duration in the high-signature group than in the low-signature group (Fig. 7C; 21 months vs. 11 months, *p* = 0.046). In the 54 BL FFPE samples, 10 genes were imputed and analyzed, including *AGR2*, *AZGP1*, *FGFR1*, *MGP*, *NDUFC2*, *RPL36A*, *RPS17*, *SLC39A6*, *TFF1* and *TFF3*. Genes with missing values or values equal to 1 across samples were removed from the analysis. The KM plot for the FFPE samples showed a similar significant improvement in mPFS duration, from 9 months to 16 months (Figure 7D; *p* = 0.035). Batch effect correction was performed when combining the FFPE and fresh tissue samples to remove technical factors (Figures S7A and S7B). Furthermore, the combined 89 samples showed a significant improvement in mPFS in the high-signature group compared to the low-signature group (Figure S7C, 19 months vs. 11 months, *p* = 0.046). These findings demonstrate the predictive capability of our panel of genes, showing an almost double prolongation of PFS and consistent validation across different sample types.

The second cohort comprised published data from Asian Korean patients [50], where bulk RNA was sequenced from 61 BL samples from patients with HR+/HER2- mBC, including 22 fresh tissue biopsies and 39 FFPE samples (Fig. 7E and Table 3). We next analyzed the upregulated gene list from Fig. 1H in the Korean cohort, and 8 genes were imputed and analyzed: *AGR2*, *AZGP1*, *MGP*, *NDUFC2*, *RPL36A*, *SLC39A6*, *TFF1*, and *TFF3*. The KM plot of these 8 genes for the Korean cohort showed a significantly longer PFS duration in the signature-high group than in the -low group (Fig. 7F; 19 months vs. 9 months, *p* < 0.0001). Similar to the first cohort, the results from the second cohort demonstrate an almost double prolongation of PFS, highlighting the predictive capability of our panel of genes. Interestingly, when we separated the EP samples from the LP samples and reanalyzed the 8 gene signatures the subsequent KM plot of EP samples did not show a significant difference in PFS between the signature-high and signature-low groups (Figure S7D). However, the KM plot of LP samples showed a significant improvement in PFS for the signature-high group compared to the signature-low group (Figure S7E; 20 months vs. 14 months, *p* = 0.0068). Since all the genes identified in our predictive signature genes (Fig. 1H) were from BL tumors with long PFS, these results suggest a robust and unbiased validation of our LP predictive signature genes.

### Broad applicability of gene signatures across clinical and biological contexts

To further assess the robustness of our predictive signatures, we analyzed de novo BL samples from patients without prior treatment and samples from patients who had received neoadjuvant or adjuvant chemotherapy before CDK4/6i therapy [50]. KM survival analysis revealed that in untreated patients, the signature-high group had significantly longer mPFS compared to the signature-low group (Figure S8A; 20 months vs. 10 months, p = 0.035). Similarly, in patients with prior chemotherapy, the signature-high group showed a significantly longer mPFS (Figure S8B; 14 months vs. 6.5 months, p = 0.017). These findings validate the predictive utility of our signatures across diverse clinical contexts and suggest their broader applicability in mBC patients.

Next, we analyzed samples from diverse tissue sites to assess the broad applicability of our predictive signatures. The signatures were analyzed separately in BL breast tumor samples from the Korean cohort, as well as in bone and liver metastatic samples from the MD Anderson cohort. KM plots revealed a significantly longer mPFS duration in the signature-high group compared to the signature-low group for breast tumors (Figure S8C; 20 months vs. 11 months, p = 0.0038). A similar trend of prolonged mPFS was observed in bone metastases (Figure S8D; 21 months vs. 12 months) and liver metastases (Figure S8E; 12 months vs. 4 months), demonstrating the broad applicability of our signatures. Furthermore, an analysis of the MD Anderson Master Database, encompassing 351 patients with HR+/HER2- mBC treated with CDK4/6is plus endocrine therapy (Table 3), demonstrated significant site-specific differences in outcomes. Patients with bone-only metastases exhibited substantially longer mPFS compared to those with liver or lung metastases (Figure S8F; 22.5 months vs. 11.5 months, p = 0.0091). These results provide critical insights into the influence of diverse metastatic site on clinical outcomes and highlight the potential utility of our signatures in stratifying patients across various metastatic sites.

We further analyzed the performance of our signatures in patients stratified by endocrine therapy, comparing those treated with CDK4/6i plus an aromatase inhibitor (e.g., letrozole) or fulvestrant. KM analysis showed that patients in the signature-high group treated with letrozole plus CDK4/6is had significantly longer mPFS (16 months vs. 11.5 months; Figure S8G; p = 0.032). A similar trend was observed in patients treated with fulvestrant plus CDK4/6i, where the mPFS for the signature-high group was 15 months versus 6 months for the signature-low group (Figure S8H). These results demonstrate that our signatures are predictive across diverse clinical contexts, including different metastatic sites and endocrine therapy regimens.

To investigate the influence of specific endocrine therapy regimens on gene expression patterns, we analyzed samples of patients receiving CDK4/6i with either letrozole or fulvestrant. In EP samples, distinct gene expression differences were observed between the two regimens (Figure S9). Genes associated with immune response, including leukotriene B4 receptor (*LTB4R*) and TLR4 interactor with leucine-rich repeats (*TRIL*), as well as the cell cycle regulator *CDKN2B* (p15) and *MDM1*, and signal transduction mediators *NOTCH2* and *MAP3K6*, were significantly increased in EP samples resistant to CDK4/6is plus fulvestrant (Figure S9). Conversely, genes associated with lipid and protein metabolism, such as pancreatic lipase (*PNLIP*) and carboxypeptidase A1 (*CPA1*), along with ribosome-associated genes (*RPL5* and *RPS24*), were significantly elevated in samples that progressed on CDK4/6is plus letrozole treatment (Figure S9). Notably, in LP samples, no significant differential gene expression was identified between the two regimens (data not shown). These results showed that specific endocrine therapy regimens distinctly influence gene expression patterns and resistance mechanisms in patients treated with CDK4/6is. Despite these differences, our predictive signature panels were validated across both treatment settings, highlighting the robustness and broad applicability of our signatures across different therapeutic regimens.

Our scRNA-seq data showed an expansion of NK and CD8^+^ T cells in responder BL samples. To validate these findings, we analyzed bulk RNA-seq data from the MD Anderson cohort using Cibersortx to deconvolute and estimate cell type proportions. The results showed that NK cell frequencies were significantly higher in responder BL and LP samples compared to EP samples (Figures S10A and S10B), consistent with our scRNA-seq findings (Fig. 3D). Longitudinal analysis of samples from the Korean cohort revealed that the LP sample exhibited higher baseline proportions of CD8^+^ T cells (25.0% vs. 15.9%) and NKT cells (8.8% vs. 6.2%) compared to the EP sample (Figures S10C and S10D). Furthermore, following progression on CDK4/6is, NKT populations expanded in LP samples (12.0% vs. 8.8% at baseline), consistent with the immune modulation observed in our scRNA-seq analysis (Figures S10D and Fig. 6H). These findings provide additional validation for our scRNA-seq results and highlight distinct immune profiles that may underpin differential responses to CDK4/6i therapy.

Taken together, these results from 2 independent cohorts of patients with HR + /HER2- mBC demonstrated the consistent and robust validation of our scRNA-seq-derived gene signatures, as well as an increase in cytotoxic NK cells, using bulk RNA-seq data from HR+/HER2- mBC patients. These findings demonstrate that our biomarker panel, derived from scRNA-seq data, is effective across diverse patient ethnicities, biopsy sources, endocrine therapies, and primary or metastatic sites. This advancement in biomarker-driven precision prediction may result in improved outcomes in patients with HR+/HER2- mBC.

## Discussion

The introduction of CDK4/6is has significantly advanced the treatment for patients with HR+/HER2- mBC. These inhibitors, when combined with endocrine therapy, have markedly improved PFS and overall survival outcomes [5–14]. However, responses to CDK4/6i therapy vary greatly, emphasizing the need to understand the mechanisms behind early versus late progression and to identify predictive biomarkers for better treatment guidance. Our study addresses this need by examining the single-cell transcriptomic landscape within the TME before and during progression on CDK4/6i therapy. The successful validation of our predictive biomarker panel in two independent cohorts of BL samples from patients with HR+/HER2- mBC highlights its potential clinical utility in optimizing patient selection and treatment outcomes.

Progression on CDK4/6is can result from intrinsic or acquired resistance mechanisms. Early progressors often exhibit intrinsic resistance due to genetic alterations in the cyclin D-CDK4/6-Rb pathway, such as amplification or mutation of CDK4/6, cyclin D, or Rb. In contrast, acquired resistance mechanisms include the activation of compensatory pathways such as FGFR1, MAPK, PI3K/AKT/mTOR, Myc, Hippo, tumor metabolism, and alterations in the TME [16, 66, 67].

Consistent with these findings, our scRNA-seq data revealed that late progressors exhibited increased expression of PI3K/AKT/mTOR and Myc-related genes, as well as enhanced inflammatory response and TNF-α pathway activity. Myc plays a central role in tumor progression by promoting cell cycle progression, metabolic reprogramming, and resistance to apoptosis, which can amplify resistance to CDK4/6is [68, 69]. The upregulation of EMT-related genes observed in our data supports the notion that EMT contributes to increased metastatic potential and resistance by facilitating cellular plasticity and evasion of therapy-induced stress [70]. Furthermore, TNF-α signaling was significantly enriched in late progressors, underscoring its role in driving chronic inflammation, reshaping the tumor immune microenvironment, and enhancing tumor survival under therapeutic pressure [29, 71].

These findings highlight the interplay between oncogenic signaling, inflammatory pathways, and immune modulation in acquired resistance to CDK4/6is. Understanding these mechanisms provides opportunities for targeted therapeutic interventions, such as combining CDK4/6is with agents targeting PI3K/AKT/mTOR, Myc, or inflammatory mediators, to overcome resistance in late-stage disease.

Research on predictive biomarkers in the context of CDK4/6i therapy for HR+/HER2- mBC has been active, yet inconsistent and reliable biomarkers remain elusive. Various investigators have proposed biomarkers based on genetic mutation profiles, including *ESR1* (ERα), *CCND1* (cyclin D1), *Rb*, and genes in the PI3K/AKT/mTOR pathway [5, 17–19, 26]. Our study advances current knowledge in several ways. First, scRNA-seq allows us to distinguish the molecular profiles of individual cells and identify more subtypes of non-tumor cells for comparison. Second, transcriptomic profiles of the TME provide insights into functional mechanisms and biomarkers that can predict outcomes with CDK4/6i treatment. Furthermore, emerging evidence suggests that the TME landscape of metastatic tumors differs from that of primary tumors [72]. Current studies often focus on primary tumor specimens, lacking insights into metastatic tumor sites and tissue-specific biomarker identification. By obtaining biopsies from different metastatic sites before (at baseline) and during CDK4/6i therapy (at progression), our data revealed dynamic changes within the TME before treatment and during disease progression, enabling the identification of both global (tissue-independent) and tissue-specific profiles and biomarkers.

TILs, including CD8^+^ T cells, have emerged as potential predictive biomarkers in HER2 + and triple-negative BC subtypes [73] and are associated with improved treatment responses, especially with immunotherapy combinations. However, their significance in HR+/HER2- BC remains uncertain due to their lower immunogenicity [74, 75]. Previous preclinical studies suggest that CDK4/6i treatment induces immunomodulation, including enhancing immunogenicity [30] and PD-L1 stability [76], indicating potential efficacy when combined with immunotherapy. Clinical trials such as PACE have demonstrated prolonged mPFS and overall survival in patients with HR+/HER2- BC when combining palbociclib with endocrine therapy and avelumab (anti-PD-L1) compared to fulvestrant alone [77]. Additionally, trials combining abemaciclib or ribociclib with pembrolizumab (anti-PD-1) ± anastrozole have shown significant efficacy, albeit with side effects such as pneumonitis and high rates of grade 3 transaminitis [78]. Clinical trials utilizing palbociclib combined with anti-PD-1 have also demonstrated enhanced efficacy [79, 80]. These clinical trial results are compelling as they provide evidence that combining CDK4/6is with immune checkpoint inhibitors can lead to better outcomes, supporting the hypothesis that immunomodulation by CDK4/6is enhances immunotherapy effectiveness.

Our scRNA-seq data showing higher levels of cytotoxic CD8^+^ T cells and NK cells in BL and LP responders support the investigation of CDK4/6is combined with immune checkpoint inhibitors in HR+/HER2- mBC and suggest a potential biomarker for identifying patients who might benefit most from this combination therapy. Validation using bulk RNA-seq data from the MD Anderson cohort confirmed that NK cell frequencies were significantly higher in BL responders and LP compared to EP samples, consistent with the immune cell dynamics observed in our scRNA-seq data. Furthermore, the analysis of longitudinal samples from the Korean cohort reinforced the importance of immune composition. The LP sample exhibited higher baseline levels of CD8^+^ T cells and NKT cells, and NKT populations expanded following CDK4/6i treatment, underscoring the immune-mediated mechanisms of late-stage progression.

These findings suggest that both tumor cell and non-tumor cell biomarkers, particularly immune cell subsets, are integral to predicting therapeutic response. While our tumor cell signature demonstrated broad validation across tissue types, the TME signatures offer a complementary perspective that warrants further exploration. Future studies should focus on integrating these biomarkers into predictive models to refine therapeutic strategies and improve outcomes for patients receiving CDK4/6is.

While both BL and LP tumors showed increased infiltrating T cells, BL T cells exhibited significantly higher expression of stress-associated genes, such as heat shock proteins, and exhaustive genes, including *LAG3*, *GNLY*, *TIGIT*, and *TNF*, than did LP T cells. Since stress genes in T cells are known to confer resistance to anti-PD1/PD-L1 immune checkpoint inhibitor treatment, which has been validated in multiple types of solid tumors [40], our data suggest a potentially favorable response to anti-PD1/PD-L1 immune therapy in tumors that progress on CDK4/6i compared to in BL tumors. Furthermore, high expression of *LAG3* and *TIGIT*, both novel immune checkpoint receptors [81], in BL tumors presents alternative immunotherapeutic opportunities for patients with HR+/HER2- mBC before CDK4/6i treatment. Significant changes in immunosuppressive regulatory T cells were not observed, likely due to the limited sample size and cell numbers.

Previous studies also highlighted significant macrophage infiltration in tumors, with increased macrophage density often associated with poor prognosis and chemotherapy resistance [82, 83]. Recent research has further revealed the transcriptional diversity and distinct ontological sources of macrophages [84]. Tumor-associated macrophages encompass anti-tumor M1 and pro-tumor M2 subtypes: M2 macrophages are linked to protumorigenic effects and repair mechanisms, and M1 macrophages exhibit anti-tumor effects and defense mechanisms [85]. Targeting macrophages for anti-cancer therapy by reprogramming M2 macrophages to M1 represents a potential therapeutic strategy. This can be achieved through interventions such as CSF-1R inhibitors, anti-CCL2 and -CCR2 antibodies, CD40 agonists, and anti-CD47 antibodies [86–88]. Furthermore, the expression of *CSF1R* and *CCL2* in M2 macrophages from EP was significantly upregulated (Figure S5F), suggesting therapeutic targets for patients with HR+/HER2- mBC who demonstrated intrinsic resistance to CDK4/6is.

Our scRNA-seq data identified key ligand-receptor (L-R) interactions, including osteopontin (SPP1-CD44) and SPP1- (ITGA4 + ITGB1) interactions in EP samples (Figs. 3F and S3A-C), and elevated midkine (MDK)-nucleolin (NCL), MDK-syndecan interactions in LP samples. Both SPP1-CD44 and MDK-NCL interactions are implicated in cancer progression and represent potential therapeutic targets. SPP1 (secreted phosphoprotein 1), also known as osteopontin, interacts with CD44 to promote tumor growth, metastasis, and immune evasion. Targeting the SPP1-CD44 has shown promise in reducing tumor progression and enhancing the efficacy of immune checkpoint inhibitors [89]. Similarly, inhibiting the MDK-NCL interaction has been proposed as a therapeutic strategy in glioblastoma and endometrial carcinoma [62, 90]. The enrichment of these interactions in EP and LP samples suggests that targeting these pathways may complement CDK4/6is by addressing L-R-mediated resistance mechanisms. Combining CDK4/6is with therapies targeting SPP1-CD44 or MDK-NCL interactions offers a promising avenue for enhancing patient outcomes. Future studies should focus on preclinical validation of these strategies and evaluating their efficacy in clinical settings.

Within tumor cells, we identified a biomarker panel from BL tumors (Fig. 1H) that is predictive of late disease progression on CDK4/6i treatment in patients with HR+/HER2- mBC, which was successfully validated in 2 independent cohorts (Figs. 7 and S7-S10). This panel of genes includes trefoil factor family proteins, TFF1 and TFF3, which have been shown to be upregulated in BL immunohistochemistry tumor samples from patients with HR+/HER2- mBC who experience late progression on CDK4/6i therapy [91]. TFF1 (also known as BC-associated peptide pS2) and TFF3 are expressed in mucosal epithelium and co-secreted with mucins in the gastrointestinal and respiratory tracts to form protective barriers [92]. As secretory proteins, serum TFF3 levels have also been proposed as a screening marker for various cancers, including breast [93], gastric [94], prostate [95], pancreatic [96], and lung [97], and cholangiocarcinoma [98]. Furthermore, our analyses of tissue-specific profiles have identified common predictive biomarkers. Interestingly, genes such as *TFF3*, *MGP*, and *IGKC* were identified across all BL responder tumor cells, irrespective of metastatic site or tissue-specific signatures. This supports their potential utility in guiding treatment decisions across a wide range of clinical contexts. We also identified pleural effusion predictive gene signatures, including *TMPRSS3*, *MS4A7*, *NAPSA*, and *IGFBP2*, while liver metastasis predictive markers included *WFDC2*, *HSPB1*, *COX6C*, *APOE*, and *TUBA1A* (Fig. 2M). These findings provide a foundation for personalized therapeutic strategies tailored to metastatic site and molecular profiles, emphasizing the importance of integrating site-specific considerations into clinical decision-making.

Our analysis of non-tumor cells, such as T cells, NK cells, and B cells, also identified *TFF3* as a consistent predictive biomarker for favorable outcomes with CDK4/6is. These results indicate that *TFF3* expression has been observed not only in BC cells but also in immune cells, including T, NK, and B cells. Whether in tumor or non-tumor cells, *TFF3* consistently exhibited upregulation in BL samples from patients who responded well to CDK4/6is.

From our signature panels, all genes were successfully imputed by Cibersortx in fresh tissue samples from the MD Anderson cohort. However, in FFPE samples from the same cohort, only 10 genes could be imputed, and in the Korean cohort, 8 genes were imputed. This discrepancy reflects differences in sequencing depth, sample resources, and overall sample quality across cohorts. Several factors may explain the failed imputation of certain genes by Cibersortx: (1) low or absent gene expression in bulk RNA-seq data, (2) variability in RNA-seq data quality, (3) lack of cell type-specific expression, (4) insufficient representation of certain genes across cell types, and (5) methodological limitations of Cibersortx in capturing complex gene expression patterns.

Furthermore, the biological differences between the cohorts may have contributed to the discrepancy. For instance, approximately 60% of the Korean cohort samples were derived from primary breast tumors, whereas the fresh biopsies from MD Anderson cohort exclusively comprised metastatic samples from bone, liver, and other sites. Despite these differences, our results demonstrated the robustness of the core biomarker genes, which consistently differentiated PFS across diverse clinical settings. This validation across multiple sample types, tissue origins, and treatment regimens underscores the broad translational applicability of our predictive signatures. These findings also provide new insights into the interplay between tumor cell heterogeneity, tissue-specific gene expression, and resistance mechanisms to CDK4/6is.

Our analysis revealed that specific endocrine therapy regimens may influence gene expression patterns and resistance mechanisms in patients treated with CDK4/6is. For instance, the analysis of differential genes expression identified the upregulation of genes associated with immune response, cell cycle regulator and signal transduction mediators in patients treated with CDK4/6is plus fulvestrant. In contrast, genes related to lipid and protein metabolism, as well as ribosome function, were significantly elevated in samples that progressed on CDK4/6is plus letrozole treatment. This suggests that the choice of endocrine therapy could modulate molecular pathways contributing to resistance, aligning with prior evidence that therapeutic context can shape tumor evolution under treatment pressure.

Notably, our predictive signature panels retained their validity across both treatment regimens, demonstrating robust performance in distinguishing PFS outcomes for patients treated with letrozole or fulvestrant in combination with CDK4/6is. These findings underscore the versatility of the signatures and their potential to guide clinical decision-making across diverse therapeutic contexts.

Our study has some limitations. First, the sample size was relatively small, reflecting the challenges in obtaining biopsies from metastatic sites of disease from real-world patients treated outside of clinical trials. The small number of longitudinal samples limits the generalizability of findings regarding site-specific progression mechanisms. Therefore, further studies involving larger cohorts are necessary to validate the observed differences in gene expression and to elucidate their functional implications for resistance mechanisms. Future research should explore whether tailoring treatment regimens to specific molecular profiles could improve outcomes for patients with CDK4/6i-resistant disease. Second, the majority of patients in our cohort received palbociclib combined with endocrine therapy, and the effects of other CDK4/6is, such as ribociclib and abemaciclib, on the TME warrant further exploration. Potential confounding factors, such as comorbidities, was not fully accounted for, which could influence the study's findings. Third, while our study highlights molecular mechanisms underlying resistance to CDK4/6is, it is limited by the absence of samples from patients receiving alternative therapies post-CDK4/6i progression. Without such samples, it is not possible to definitively determine whether the observed molecular changes are unique to CDK4/6i resistance or shared across other therapeutic settings. To partially address this limitation, we analyzed both de novo patients and those with prior chemotherapy, demonstrating that our predictive signatures are effective in differentiating PFS in both contexts. However, future studies should expand to include samples from patients on alternative therapies to comprehensively evaluate therapy-specific molecular changes and validate our findings in diverse treatment settings.

## Conclusions

In summary, our study provided valuable insights into the transcriptomic landscape of HR+/HER2- mBC and validated a biomarker panel for predicting late progression on CDK4/6i therapy using bulk RNA-seq data from 2 independent cohorts. By identifying predictive molecular biomarkers in both tumor and non-tumor cell types within the TME, we emphasized the importance of precision medicine in developing personalized treatments. These findings could help guide treatment decisions and improve outcomes for patients with HR+/HER2- mBC.

## Supplementary Information

Supplementary Material 1.

Supplementary Material 2.

Supplementary Material 3.

Supplementary Material 4.

## Data Availability

Single-cell RNA-seq data are available on GEO (Accession number: GSE262288). Bulk RNA-seq data from fresh tissues are on GEO (Accession number: GSE274139), and bulk RNA-seq data from FFPE samples are also on GEO (Accession number: GSE274141). All original code is on GitHub and is publicly available as of the date of publication. DOIs: https://github.com/pengyang0411/CDK4.
