# Supplementary material for: Single-cell RNA sequencing identifies molecular biomarkers predicting late progression to CDK4/6 inhibition in patients with HR+/HER2- metastatic breast cancer
Source: Mol Cancer. 2025 Feb 15;24:48. doi: 10.1186/s12943-025-02226-9 (PMC11829392; doi:10.1186/s12943-025-02226-9)
Supplement: Supplementary file 1 — Supplementary Material 1. [file 12943_2025_2226_MOESM1_ESM.pdf]

Single-Cell RNA Sequencing Identifies Molecular Biomarkers Predicting Late Progression to CDK4/6 Inhibition in Patients with HR+/HER2- Metastatic Breast Cancer

Linjie Luo[1,7,**], Peng Yang[2,3,7], Sofia Mastoraki[1], Xiayu Rao[4], Yan Wang[1], Nicole M. Kettner[1], Akshara Singareeka Raghavendra[5], Debasish Tripathy[5], Senthil Damodaran[5], Kelly K. Hunt[6], Jing Wang[4], Ziyi Li[3], Khandan Keyomarsi[1,*]

[1]Department of Experimental Radiation Oncology, The University of Texas MD Anderson Cancer Center, Houston, TX, USA

[2]Department of Statistics, Rice University, Houston, TX, USA

[3]Department of Biostatistics, The University of Texas MD Anderson Cancer Center, Houston, TX, USA

[4]Department of Bioinformatics and Computational Biology, The University of Texas MD Anderson Cancer Center, Houston, TX, USA

[5]Department of Breast Medical Oncology, Division of Cancer Medicine, The University of MD Anderson Cancer Center, Houston, TX, USA

[6]Department of Breast Surgical Oncology, The University of Texas MD Anderson Cancer Center, Houston, TX, USA

[7]These authors contributed equally

*Correspondence: kkeyomar@mdanderson.org

**Co-correspondence: lluo2@mdanderson.org

Figure S1. UMAP of each sample and differentially expressed gene analysis on tumor cells

(A) Distinguishing tumor versus non-tumor cells via InferCNV across each sample.

(B) UMAP plot across 18 samples in the embedding space.

(C) Volcano plot showing differentially expressed genes between the BL and EP samples.

(D) Volcano plot showing differentially expressed genes between the BL and LP samples.

(E) Volcano plot showing differentially expressed genes between the EP and LP samples.

(F) MP analysis heatmap showing the normalized module scores across group comparisons.

Figure S1

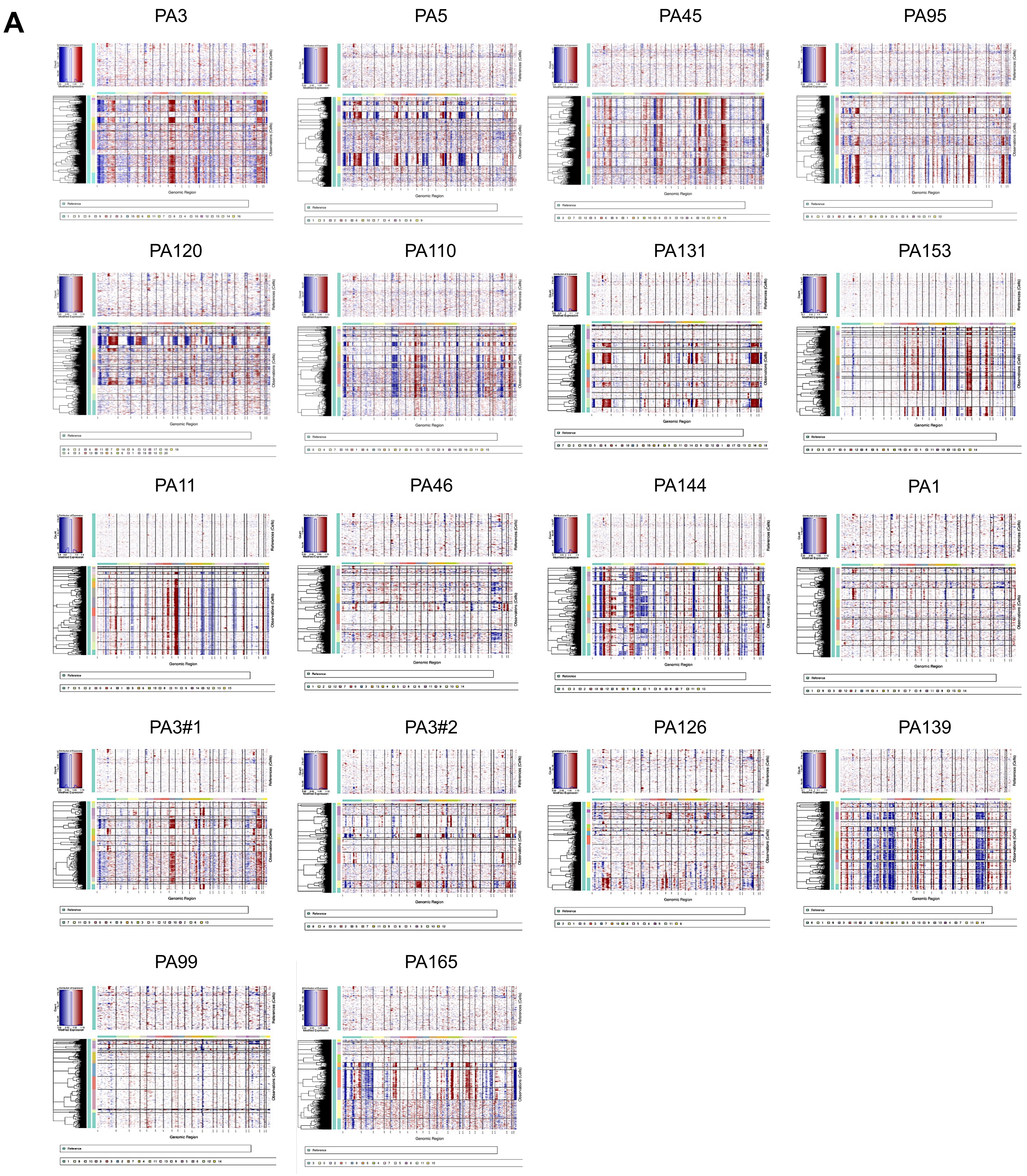
A
PA3
PA5
PA45
PA95
PA120
PA110
PA131
PA153
PA11
PA46
PA144
PA1
PA3#1
PA3#2
PA126
PA139
PA99
PA165

Figure S1

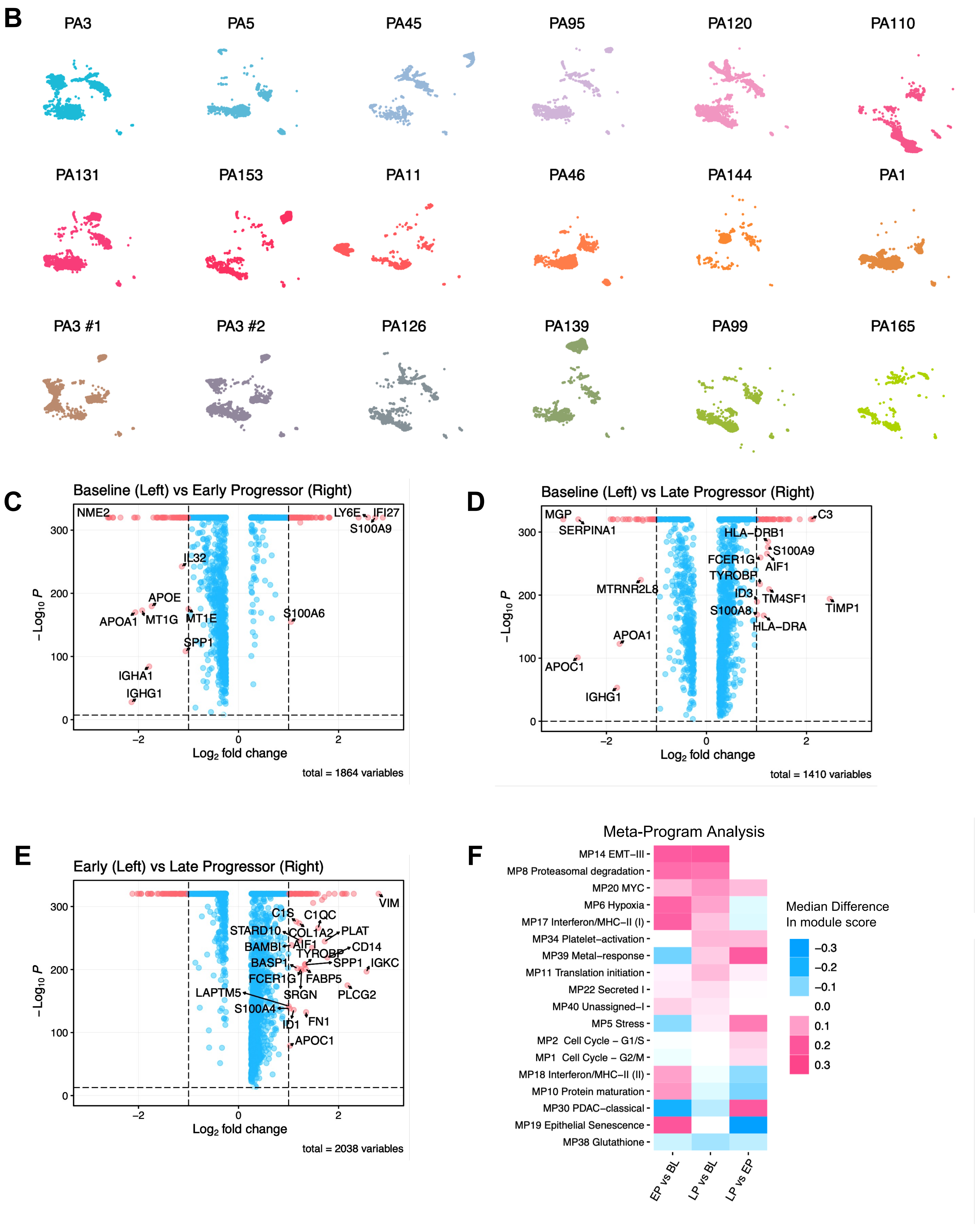
B
PA3
PA5
PA45
PA95
PA120
PA110
PA131
PA153
PA11
PA46
PA144
PA1
PA3 #1
PA3 #2
PA126
PA139
PA99
PA165
C
Baseline (Left) vs Early Progressor (Right)
NME2
LY6E
IFI27
S100A9
IL32
APOE
APOA1
MT1G
MT1E
S100A6
SPP1
IGHA1
IGHG1
−Log10 P
Log2 fold change
total = 1864 variables
D
Baseline (Left) vs Late Progressor (Right)
MGP
C3
SERPINA1
HLA−DRB1
S100A9
FCER1G
AIF1
TYROBP
MTRNR2L8
ID3
TM4SF1
TIMP1
S100A8
HLA−DRA
APOA1
APOC1
IGHG1
total = 1410 variables
E
Early (Left) vs Late Progressor (Right)
VIM
C1S
C1QC
STARD10
COL1A2
PLAT
BAMBI
AIF1
CD14
TYROBP
BASP1
SPP1
IGKC
FCER1G
FABP5
LAPTM5
SRGN
PLCG2
S100A4
ID1
FN1
APOC1
total = 2038 variables
F
Meta-Program Analysis
MP14 EMT−III
MP8 Proteasomal degradation
MP20 MYC
MP6 Hypoxia
MP17 Interferon/MHC−II (I)
MP34 Platelet−activation
MP39 Metal−response
MP11 Translation initiation
MP22 Secreted I
MP40 Unassigned−I
MP5 Stress
MP2 Cell Cycle – G1/S
MP1 Cell Cycle – G2/M
MP18 Interferon/MHC−II (II)
MP10 Protein maturation
MP30 PDAC−classical
MP19 Epithelial Senescence
MP38 Glutathione
EP vs BL
LP vs BL
LP vs EP
Median Difference
In module score
−0.3
−0.2
−0.1
0.0
0.1
0.2
0.3

Figure S2. Tumor cell analysis based on metastatic site

(A) Heatmap showing the expression of top 10 differentially expressed genes, stratified by different sample statuses, annotated by groups and metastatic sites.

(B) Volcano plot showing differentially expressed genes between the pleural effusion (left) and liver metastases (right) in BL samples.

(C) Volcano plot showing differentially expressed genes between the pleural effusion (left) and liver metastasis (right) in LP samples.

(D) Dot plot displaying the top 10 differentially expressed genes among BL, EP, and LP samples from pleural effusion.

(E) Dot plot displaying the top 15 differentially expressed genes between BL and LP samples from liver metastases.

Figure S2

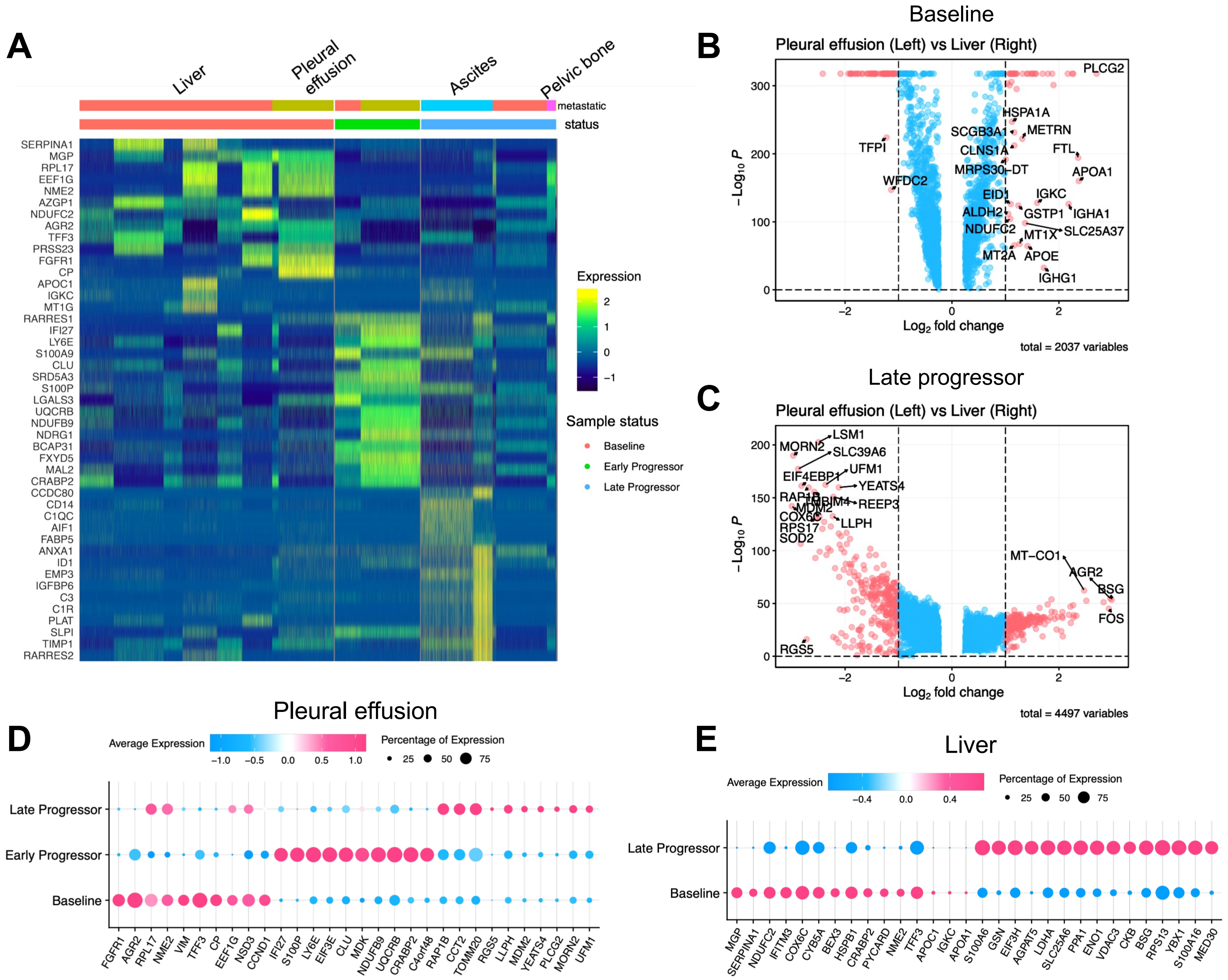

A
Liver
Pleural effusion
Ascites
Pelvic bone
metastatic
status
Expression
Sample status
Baseline
Early Progressor
Late Progressor
B
Baseline
Pleural effusion (Left) vs Liver (Right)
Log2 fold change
total = 2037 variables
C
Late progressor
Pleural effusion (Left) vs Liver (Right)
Log2 fold change
total = 4497 variables
D
Pleural effusion
Average Expression
Percentage of Expression
Late Progressor
Early Progressor
Baseline
E
Liver
Average Expression
Percentage of Expression
Late Progressor
Baseline

Figure S3. Non-tumor cells of minor-annotation cell subtype analysis

(A) Chord diagram showing ligand-receptor (L-R) interactions between 2 cell types from BL samples.

(B) Chord diagram showing L-R interactions between 2 cell types from EP samples.

(C) Chord diagram showing L-R interactions between non-tumor cells from LP samples.

(D) UMAP plots of minor non-tumor cell types stratified by different sample statuses (BL, EP, and LP).

(E) Dot plot showing the marker gene expression for each minor non-tumor cell type in (A).

(F) Cell numbers for each non-tumor cell type.

(G) Cell fraction of double-negative T (dnT) cells, gamma delta T (gdT) cells, and mucosal-associated invariant T (MAIT) cell subtypes in BL, EP, and LP samples.

Figure S3

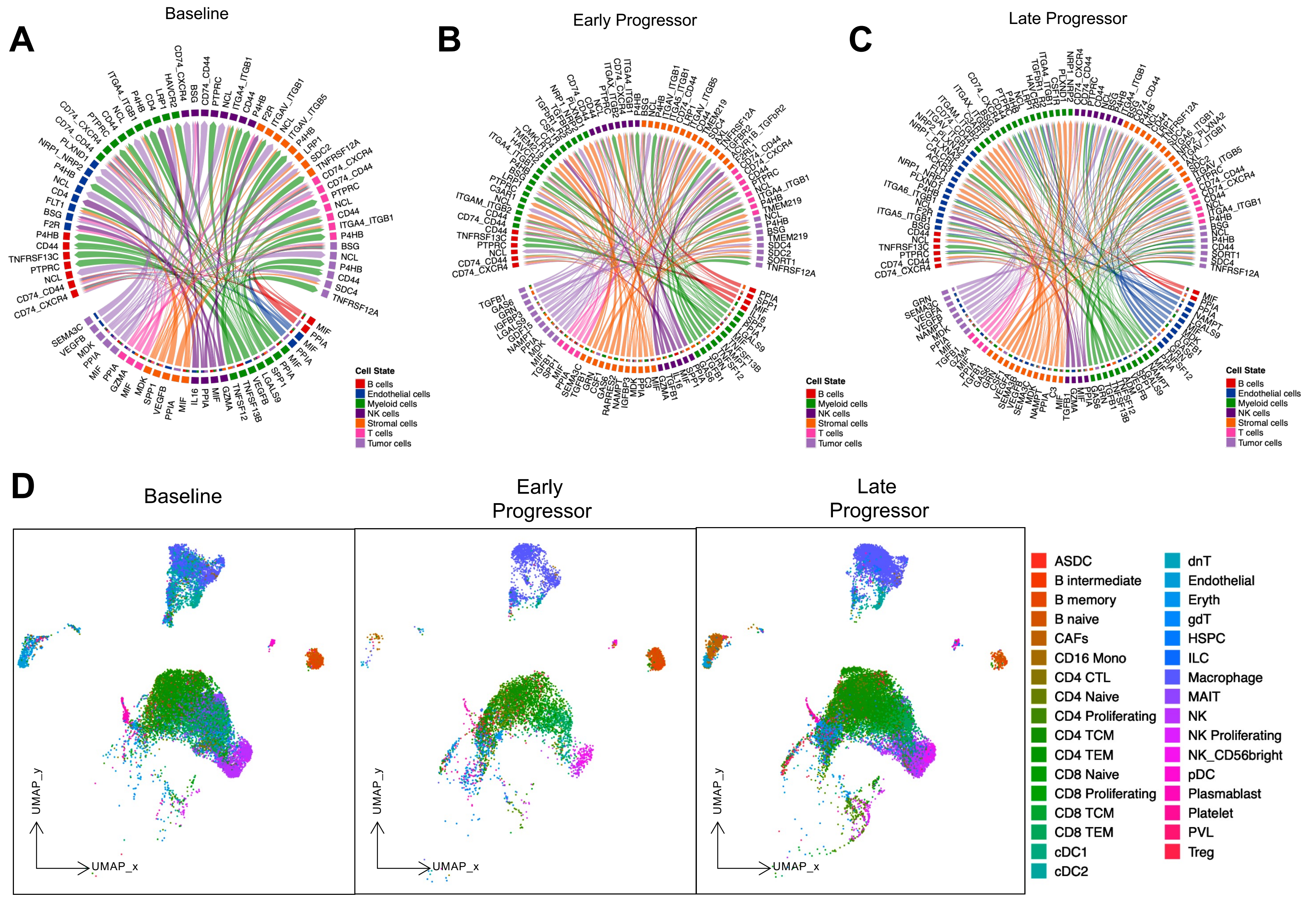

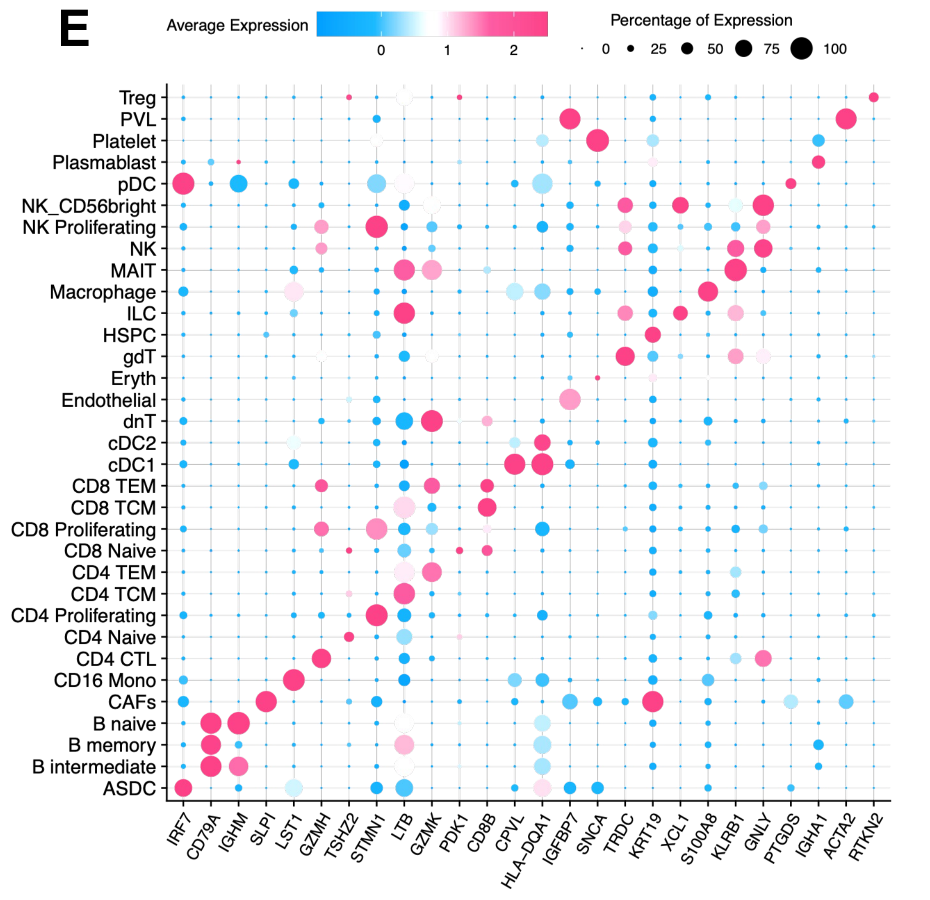

F

Cell type	Cell #	Cell type	Cell #	Cell type	Cell #	Cell type	Cell #
ASDC	4	CD4 TCM	11601	Endothelial	360	NK_CD56bright	743
B intermediate	282	CD4 TEM	1717	Eryth	1932	pDC	333
B memory	539	CD8 Naive	1096	gdT	788	Plasmablast	779
B naive	1013	CD8 Proliferating	62	HSPC	324	Platelet	2
CAFs	677	CD8 TCM	630	ILC	28	PVL	35
CD16 Mono	164	CD8 TEM	6460	Macrophage	6393	Treg	897
CD4 CTL	733	cDC1	50	MAIT	1141		
CD4 Naive	1332	cDC2	2282	NK	3641		
CD4 Proliferating	272	dnT	43	NK Proliferating	210		

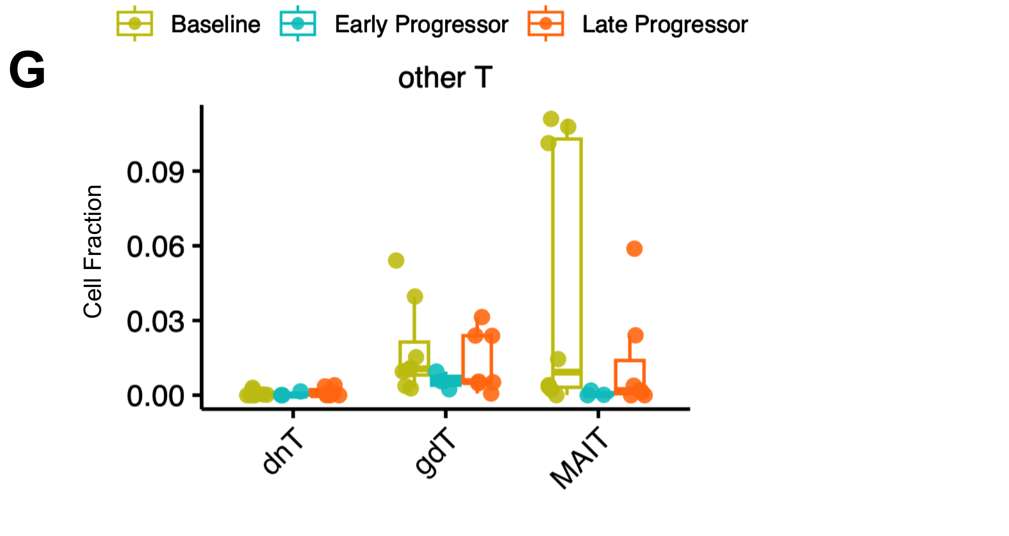

Figure S4. T cell analysis based on metastatic site

(A) Expression of stress genes in $CD4^+$ T cells from BL, EP, and LP samples.

(B) Module score analysis of $CD4^+$ T_{STR} cells among BL, EP, and LP samples.

(C) Expression of stress genes in $CD8^+$ T cells from BL, EP, and LP samples.

(D) Module score analysis of $CD8^+$ T_{STR} cells among BL, EP, and LP samples.

(E) UMAP plots of 12 different subtypes of T cells stratified by metastatic site, including ascites, liver, pelvic bone, and pleural effusion.

(F) Bar plot showing the relative fraction of T cell subtypes for each sample, annotated by group and metastatic site.

(G) Dot plot displaying the top 15 differentially expressed genes between pleural effusion and liver metastasis from BL T cells.

(H) Dot plot displaying the top 15 differentially expressed genes between pleural effusion and liver metastasis from LP T cells.

Figure S4

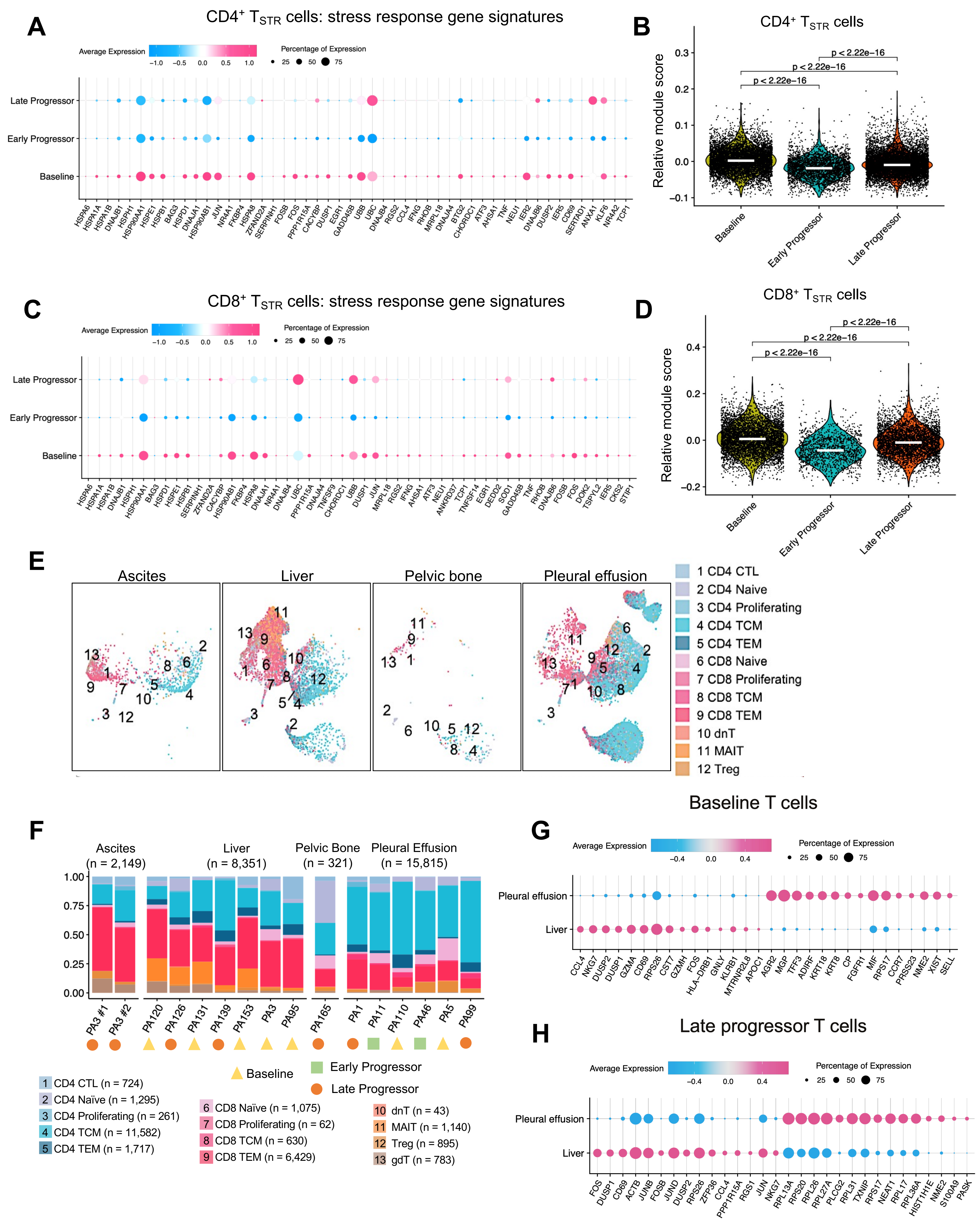

A
CD4+ TSTR cells: stress response gene signatures
Average Expression
−1.0 −0.5 0.0 0.5 1.0
Percentage of Expression
25 50 75
Late Progressor
Early Progressor
Baseline
HSPA6 HSPA1A HSPA1B DNAJB1 HSPH1 HSP90AA1 HSPE1 HSPB1 BAG3 HSPD1 DNAJA1 HSP90AB1 JUN NR4A1 FKBP4 HSPA8 ZFAND2A SERPINH1 FOSB FOS PPP1R15A CACYBP DUSP1 EGR1 GADD45B UBB UBC DNAJB4 RGS2 CCL4 IFNG RHOB MRPL18 DNAJA4 BTG2 CHORDC1 ATF3 AHSA1 TNF NEU1 IER2 DNAJB6 DUSP2 IER5 CD69 SERTAD1 ANXA1 KLF6 NR4A2 TCP1
B
CD4+ TSTR cells
Relative module score
p < 2.22e−16
p < 2.22e−16
p < 2.22e−16
Baseline
Early Progressor
Late Progressor
C
CD8+ TSTR cells: stress response gene signatures
Average Expression
−1.0 −0.5 0.0 0.5 1.0
Percentage of Expression
25 50 75
Late Progressor
Early Progressor
Baseline
HSPA6 HSPA1A HSPA1B DNAJB1 HSPH1 HSP90AA1 BAG3 HSPD1 HSPE1 HSPB1 SERPINH1 ZFAND2A CACYBP HSP90AB1 FKBP4 HSPA8 DNAJA1 NR4A1 DNAJB4 UBC PPP1R15A DNAJA4 TNFSF9 CHORDC1 UBB DUSP1 JUN MRPL18 RGS2 IFNG AHSA1 ATF3 NEU1 ANKRD37 TCP1 TNFSF14 EGR1 DEDD2 SOD1 GADD45B TNF RHOB DNAJB6 FOSB FOS DOK2 TSPYL2 IER5 CKS2 STIP1
D
CD8+ TSTR cells
Relative module score
p < 2.22e−16
p < 2.22e−16
p < 2.22e−16
Baseline
Early Progressor
Late Progressor
E
Ascites
Liver
Pelvic bone
Pleural effusion
1 CD4 CTL
2 CD4 Naive
3 CD4 Proliferating
4 CD4 TCM
5 CD4 TEM
6 CD8 Naive
7 CD8 Proliferating
8 CD8 TCM
9 CD8 TEM
10 dnT
11 MAIT
12 Treg
F
Ascites (n = 2,149)
Liver (n = 8,351)
Pelvic Bone (n = 321)
Pleural Effusion (n = 15,815)
PA3 #1 PA3 #2 PA120 PA126 PA131 PA139 PA153 PA3 PA95 PA165 PA1 PA11 PA110 PA46 PA5 PA99
Baseline
Early Progressor
Late Progressor
1 CD4 CTL (n = 724)
2 CD4 Naïve (n = 1,295)
3 CD4 Proliferating (n = 261)
4 CD4 TCM (n = 11,582)
5 CD4 TEM (n = 1,717)
6 CD8 Naïve (n = 1,075)
7 CD8 Proliferating (n = 62)
8 CD8 TCM (n = 630)
9 CD8 TEM (n = 6,429)
10 dnT (n = 43)
11 MAIT (n = 1,140)
12 Treg (n = 895)
13 gdT (n = 783)
G
Baseline T cells
Average Expression
−0.4 0.0 0.4
Percentage of Expression
25 50 75
Pleural effusion
Liver
CCL4 NKG7 DUSP2 DUSP1 GZMA CD69 RPS26 CST7 GZMH FOS HLA-DRB1 GNLY KLRB1 MTRNR2L8 APOC1 AGR2 MGP TFF3 ADIRF KRT18 KRT8 CP FGFR1 MIF RPS17 CCR7 PRSS23 NME2 XIST SELL
H
Late progressor T cells
Average Expression
−0.4 0.0 0.4
Percentage of Expression
25 50 75
Pleural effusion
Liver
FOS DUSP1 CD69 ACTB JUNB FOSB JUND DUSP2 RPS26 ZFP36 CCL4 PPP1R15A RGS1 JUN NKG7 RPL13A RPS20 RPL26 RPL27A PLCG2 RPL31 TXNIP RPS17 NEAT1 RPL17 RPL36A HIST1H1E NME2 S100A9 PASK

Figure S5. Analysis of other non-tumor cells

(A) t-SNE plot of 13 different subtypes of myeloid cells in the embedding space.

(B) Dot plot showing the expression of M1 and M2 macrophage gene signatures across each cluster from (A).

(C) Dot plots displaying the top 5 differentially expressed genes in each cluster from (A).

(D) UMAP plot of B cell populations stratified by 4 different cell subtypes.

(E) Comparison of fractions of different B cell subtypes among BL, EP, and LP samples.

(F) Violin plots showing significant changes of expression of *CSF1R* and *CCL2* among BL, EP, and LP samples. *, $p < 0.05$; **, $p < 0.01$; ***, $p < 0.001$; ****, $p < 0.0001$.

Figure S5

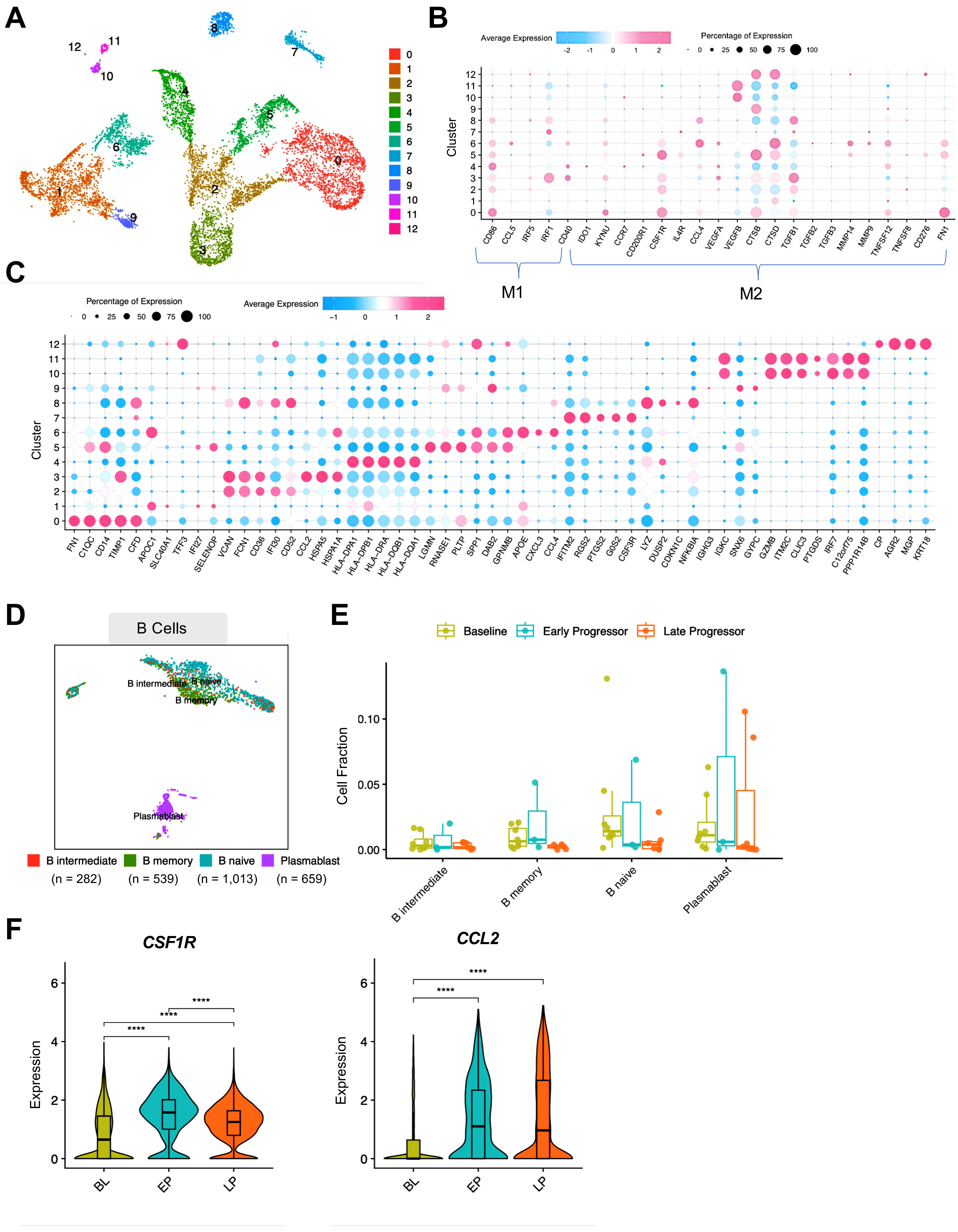

A
B
Average Expression
Percentage of Expression
Cluster
CD86 CCL5 IRF5 IRF1 CD40 IDO1 KYNU CCR7 CD200R1 CSF1R IL4R CCL4 VEGFA VEGFB CTSB CTSD TGFB1 TGFB2 TGFB3 MMP14 MMP9 TNFSF12 TNFSF8 CD276 FN1
M1
M2
C
Percentage of Expression
Average Expression
Cluster
FN1 C1QC CD14 TIMP1 CFD APOC1 SLC40A1 TFF3 IFI27 SELENOP VCAN FCN1 CD36 IFI30 CD52 CCL2 HSPA5 HSPA1A HLA-DPA1 HLA-DPB1 HLA-DRA HLA-DQB1 HLA-DQA1 LGMN RNASE1 PLTP SPP1 DAB2 GPNMB APOE CXCL3 CCL4 IFITM2 RGS2 PTGS2 G0S2 CSF3R LYZ DUSP2 CDKN1C NFKBIA IGHG3 IGKC SNX6 GYPC GZMB ITM2C CLIC3 PTGDS IRF7 C12orf75 PPP1R14B CP AGR2 MGP KRT18
D
B Cells
B intermediate
B naive
B memory
Plasmablast
B intermediate (n = 282)
B memory (n = 539)
B naive (n = 1,013)
Plasmablast (n = 659)
E
Baseline
Early Progressor
Late Progressor
Cell Fraction
0.10
0.05
0.00
B intermediate
B memory
B naive
Plasmablast
F
CSF1R
CCL2
Expression
****
BL
EP
LP

Figure S6. Longitudinal biopsy analysis

(A) Heatmap generated from MP analysis displaying the median module score differences from tumor cells between each sample.

(B) Ligand-receptor (L-R) interaction analysis conducted using CellChat in the BL sample PA3.

(C) Chord diagram illustrating L-R interactions between 2 cell types from the BL sample PA3.

(D) Chord diagram illustrating L-R interactions between 2 cell types from LP samples.

(E) UMAP plot of non-tumor cells with minor non-tumor cell types of annotation and Harmony integration.

(F) Alluvial plot showing the dynamic changes of non-tumor cell fractions across 3 longitudinal samples. The color legend is the same as in panel (E).

Figure S6

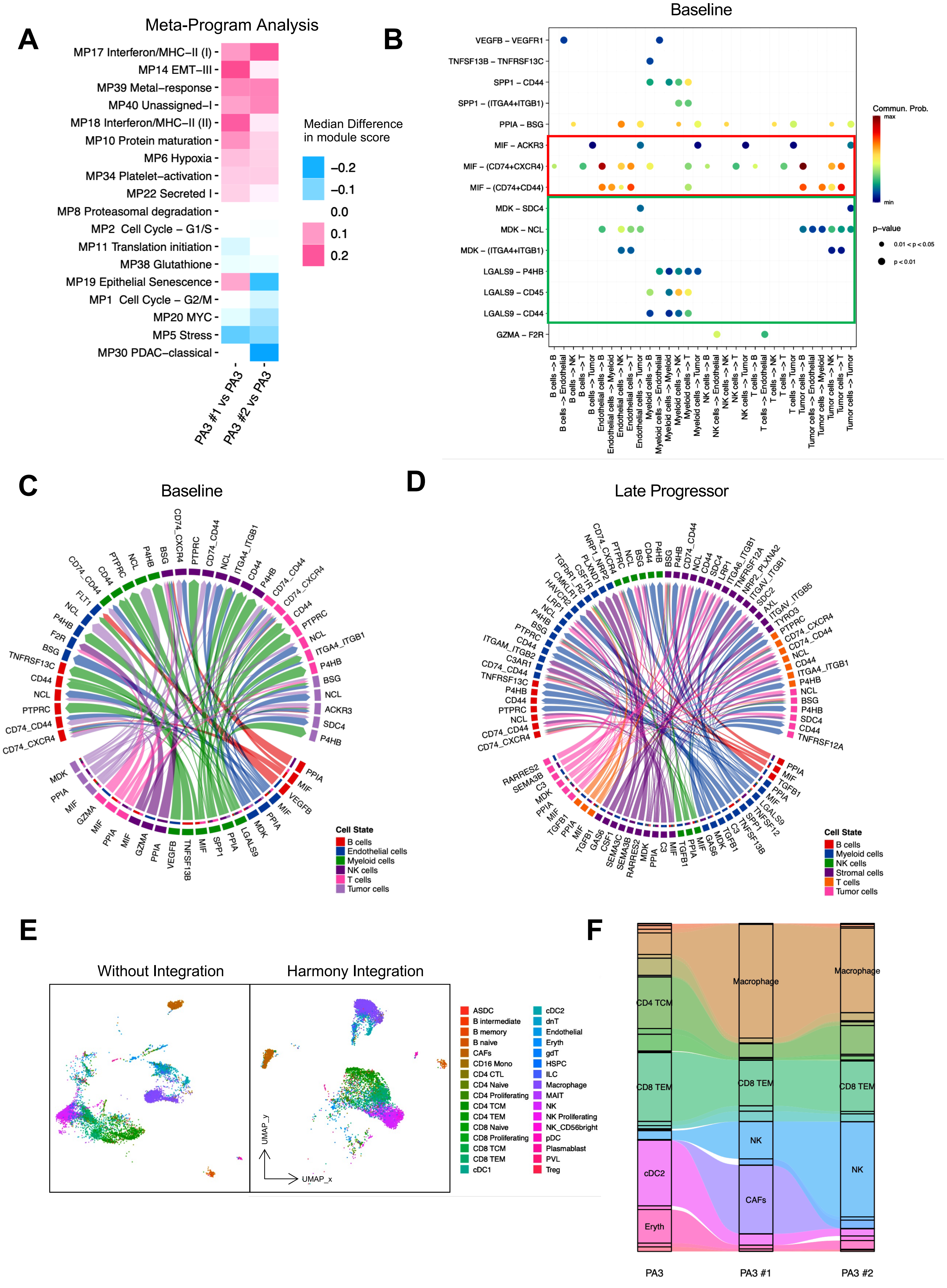
A
Meta-Program Analysis
MP17 Interferon/MHC−II (I)
MP14 EMT−III
MP39 Metal−response
MP40 Unassigned−I
MP18 Interferon/MHC−II (II)
MP10 Protein maturation
MP6 Hypoxia
MP34 Platelet−activation
MP22 Secreted I
MP8 Proteasomal degradation
MP2 Cell Cycle − G1/S
MP11 Translation initiation
MP38 Glutathione
MP19 Epithelial Senescence
MP1 Cell Cycle − G2/M
MP20 MYC
MP5 Stress
MP30 PDAC−classical
PA3 #1 vs PA3
PA3 #2 vs PA3
Median Difference in module score
−0.2
−0.1
0.0
0.1
0.2
B
Baseline
VEGFB − VEGFR1
TNFSF13B − TNFRSF13C
SPP1 − CD44
SPP1 − (ITGA4+ITGB1)
PPIA − BSG
MIF − ACKR3
MIF − (CD74+CXCR4)
MIF − (CD74+CD44)
MDK − SDC4
MDK − NCL
MDK − (ITGA4+ITGB1)
LGALS9 − P4HB
LGALS9 − CD45
LGALS9 − CD44
GZMA − F2R
Commun. Prob.
max
min
p−value
0.01 < p < 0.05
p < 0.01
C
Baseline
Cell State
B cells
Endothelial cells
Myeloid cells
NK cells
T cells
Tumor cells
D
Late Progressor
Cell State
B cells
Myeloid cells
NK cells
Stromal cells
T cells
Tumor cells
E
Without Integration
Harmony Integration
UMAP_y
UMAP_x
ASDC
B intermediate
B memory
B naive
CAFs
CD16 Mono
CD4 CTL
CD4 Naive
CD4 Proliferating
CD4 TCM
CD4 TEM
CD8 Naive
CD8 Proliferating
CD8 TCM
CD8 TEM
cDC1
cDC2
dnT
Endothelial
Eryth
gdT
HSPC
ILC
Macrophage
MAIT
NK
NK Proliferating
NK_CD56bright
pDC
Plasmablast
PVL
Treg
F
CD4 TCM
CD8 TEM
cDC2
Eryth
Macrophage
NK
CAFs
PA3
PA3 #1
PA3 #2

Figure S7. Predictive biomarker signature validation

(A) Log2 expression of signature genes across all MDACC samples before the batch effect correction.

(B) Log2 expression of signature genes across all MDACC samples after the batch effect correction.

(C) KM plot illustrating median PFS in the signature-high group compared to the -low group of 89 combined FFPE and fresh tissue samples from the MD Anderson cohort.

(D) KM plot indicating median PFS in the signature-high group compared to the -low group of 19 EP samples from the Korean cohort.

(E) KM plot indicating median PFS in the signature-high group compared to the -low group of 42 LP samples from the Korean cohort.

Figure S7

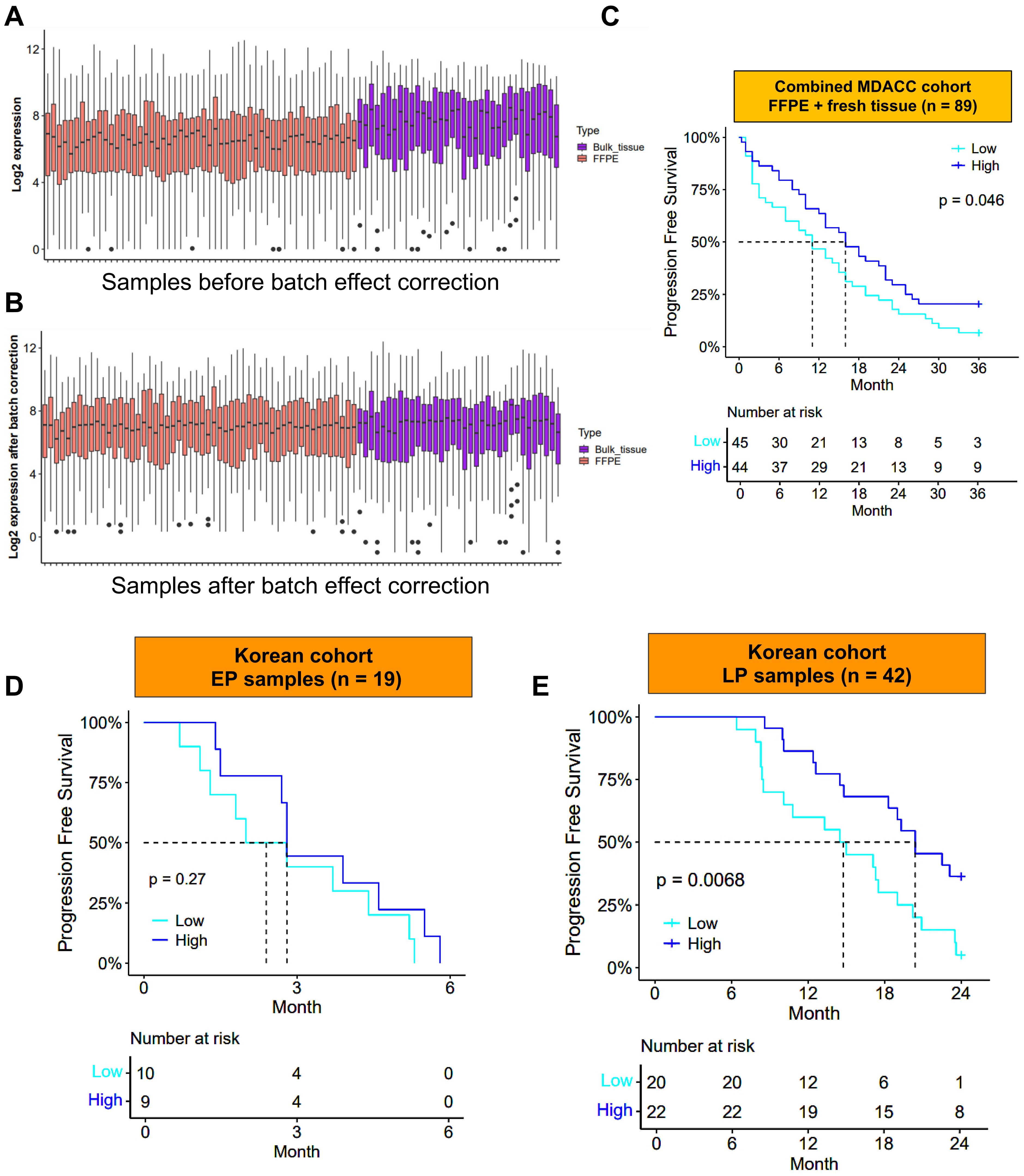
A
Log2 expression
12
8
4
0
Type
Bulk_tissue
FFPE
Samples before batch effect correction
B
Log2 expression after batch correction
12
8
4
0
Type
Bulk_tissue
FFPE
Samples after batch effect correction
C
Combined MDACC cohort
FFPE + fresh tissue (n = 89)
Progression Free Survival
100%
75%
50%
25%
0%
Low
High
p = 0.046
0 6 12 18 24 30 36
Month
Number at risk
Low 45 30 21 13 8 5 3
High 44 37 29 21 13 9 9
0 6 12 18 24 30 36
Month
Korean cohort
EP samples (n = 19)
D
Progression Free Survival
100%
75%
50%
25%
0%
p = 0.27
Low
High
0 3 6
Month
Number at risk
Low 10 4 0
High 9 4 0
0 3 6
Month
Korean cohort
LP samples (n = 42)
E
Progression Free Survival
100%
75%
50%
25%
0%
p = 0.0068
Low
High
0 6 12 18 24
Month
Number at risk
Low 20 20 12 6 1
High 22 22 19 15 8
0 6 12 18 24
Month

Figure S8. Broad applicability of gene signatures across clinical and biological contexts

(A) KM plot illustrating PFS in the signature-high group compared to the -low group in 22 *de novo* (untreated) samples from the Korean cohort from the Korean cohort.

(B) KM plot illustrating PFS in the signature-high group compared to the -low group in 39 samples from patients who received neoadjuvant or adjuvant chemotherapy in the Korean cohort.

(C) KM plot illustrating PFS in the signature-high group compared to the -low group in 36 primary breast tumors from the Korean cohort.

(D) KM plot indicating PFS in the signature-high group compared to the -low group of 33 bone metastasis tissue samples from the MD Anderson cohort.

(E) KM plot indicating PFS in the signature-high group compared to the -low group of 23 liver metastasis tissue samples from the MD Anderson cohort.

(F) KM plot indicating PFS in stratified samples from different metastatic sites, including 223 bone, 62 liver, 38 lung, and 18 pleural effusions, from the master database of the MD Anderson cohort. Censored data points indicate patients who are still undergoing treatment and have not yet developed progression.

(G) KM plot indicating PFS in the signature-high group compared to the -low group in 36 samples treated with CDK4/6i plus aromatase inhibitor (e.g., letrozole) from the MD Anderson cohort.

(H) KM plot indicating PFS in the signature-high group compared to the -low group in 16 samples treated with CDK4/6i plus fulvestrant from the MD Anderson cohort. Log-rank test p-values are displayed.

Figure S8

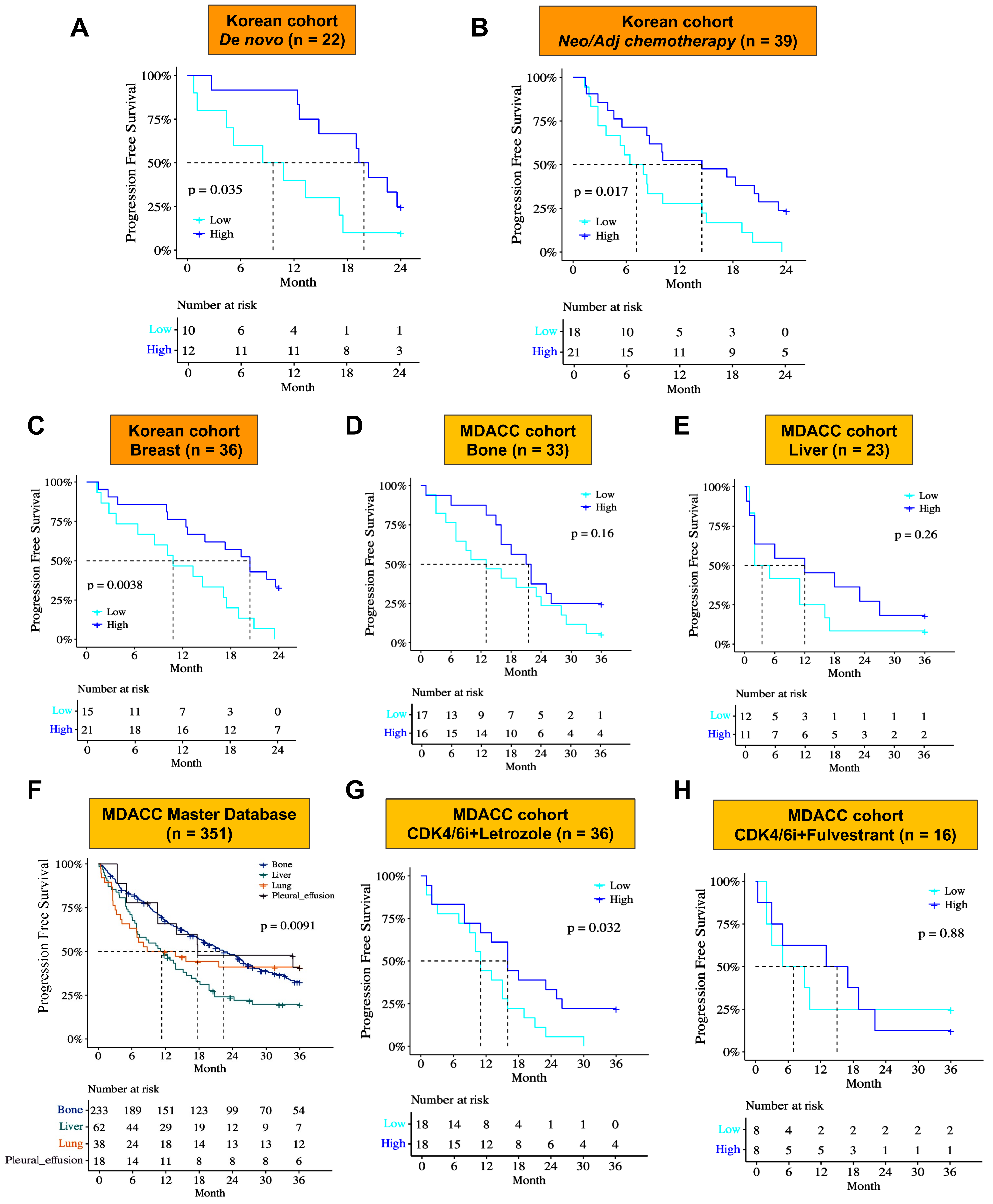
A
Korean cohort
De novo (n = 22)
Progression Free Survival
p = 0.035
Low
High
Month
Number at risk
Low 10 6 4 1 1
High 12 11 11 8 3
B
Korean cohort
Neo/Adj chemotherapy (n = 39)
p = 0.017
Low 18 10 5 3 0
High 21 15 11 9 5
C
Korean cohort
Breast (n = 36)
p = 0.0038
Low 15 11 7 3 0
High 21 18 16 12 7
D
MDACC cohort
Bone (n = 33)
p = 0.16
Low 17 13 9 7 5 2 1
High 16 15 14 10 6 4 4
E
MDACC cohort
Liver (n = 23)
p = 0.26
Low 12 5 3 1 1 1 1
High 11 7 6 5 3 2 2
F
MDACC Master Database
(n = 351)
Bone
Liver
Lung
Pleural_effusion
p = 0.0091
Bone 233 189 151 123 99 70 54
Liver 62 44 29 19 12 9 7
Lung 38 24 18 14 13 13 12
Pleural_effusion 18 14 11 8 8 8 6
G
MDACC cohort
CDK4/6i+Letrozole (n = 36)
p = 0.032
Low 18 14 8 4 1 1 0
High 18 15 12 8 6 4 4
H
MDACC cohort
CDK4/6i+Fulvestrant (n = 16)
p = 0.88
Low 8 4 2 2 2 2 2
High 8 5 5 3 1 1 1

Figure S9. Differential gene expression between CDK4/6i plus letrozole and fulvestrant in EP samples from the FFPE MD Anderson cohort

(A) Heatmap showing the expression of the top 20 differentially expressed genes, stratified by endocrine treatment regimen in EP patients from the FFPE MD Anderson Cohort. CDK4/6i plus letrozole (n = 9) and CDK4/6i plus fulvestrant (n = 7).

Figure S9

A

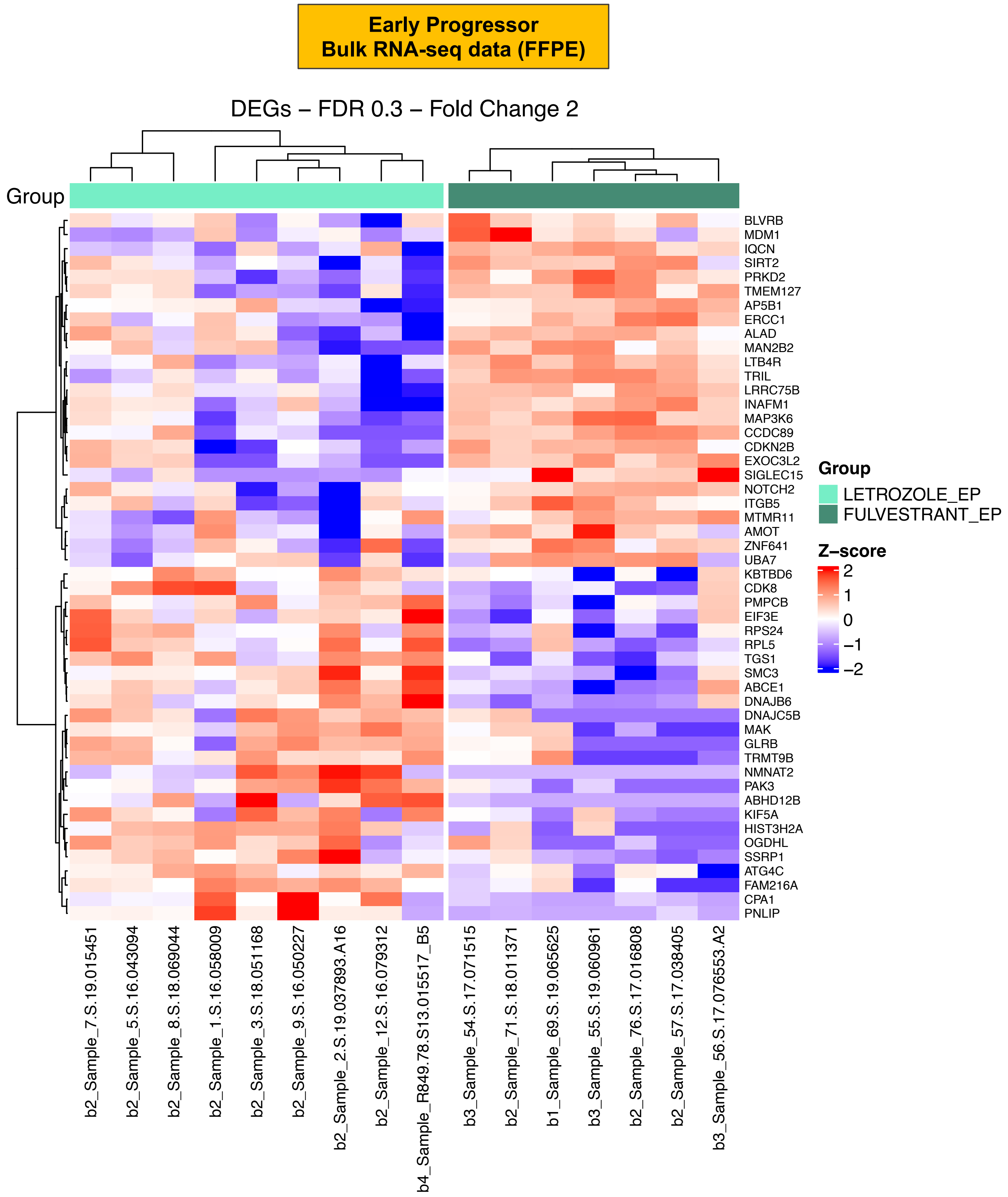

Early Progressor
Bulk RNA-seq data (FFPE)
DEGs – FDR 0.3 – Fold Change 2
Group
BLVRB
MDM1
IQCN
SIRT2
PRKD2
TMEM127
AP5B1
ERCC1
ALAD
MAN2B2
LTB4R
TRIL
LRRC75B
INAFM1
MAP3K6
CCDC89
CDKN2B
EXOC3L2
SIGLEC15
NOTCH2
ITGB5
MTMR11
AMOT
ZNF641
UBA7
KBTBD6
CDK8
PMPCB
EIF3E
RPS24
RPL5
TGS1
SMC3
ABCE1
DNAJB6
DNAJC5B
MAK
GLRB
TRMT9B
NMNAT2
PAK3
ABHD12B
KIF5A
HIST3H2A
OGDHL
SSRP1
ATG4C
FAM216A
CPA1
PNLIP
b2_Sample_7.S.19.015451
b2_Sample_5.S.16.043094
b2_Sample_8.S.18.069044
b2_Sample_1.S.16.058009
b2_Sample_3.S.18.051168
b2_Sample_9.S.16.050227
b2_Sample_2.S.19.037893.A16
b2_Sample_12.S.16.079312
b4_Sample_R849.78.S13.015517_B5
b3_Sample_54.S.17.071515
b2_Sample_71.S.18.011371
b1_Sample_69.S.19.065625
b3_Sample_55.S.19.060961
b2_Sample_76.S.17.016808
b2_Sample_57.S.17.038405
b3_Sample_56.S.17.076553.A2
Group
LETROZOLE_EP
FULVESTRANT_EP
Z–score
2
1
0
−1
−2

Figure S10. Predictive cell type signature validation

(A) Bar plot showing the relative fractions of tumor and major non-tumor cell types in samples from the MD Anderson Cohort.

(B) Cell fraction frequencies of major non-tumor cell types across sample statuses.

(C) Alluvial plot showing the dynamic changes in major non-tumor cell subtype fractions across longitudinal samples from an EP patient (BRO7F.061) from the Korean cohort.

(D) Alluvial plot illustrating the dynamic changes in major non-tumor cell subtype fractions across longitudinal samples from an LP patient (BRO7F.021) from the Korean cohort.

Figure S10

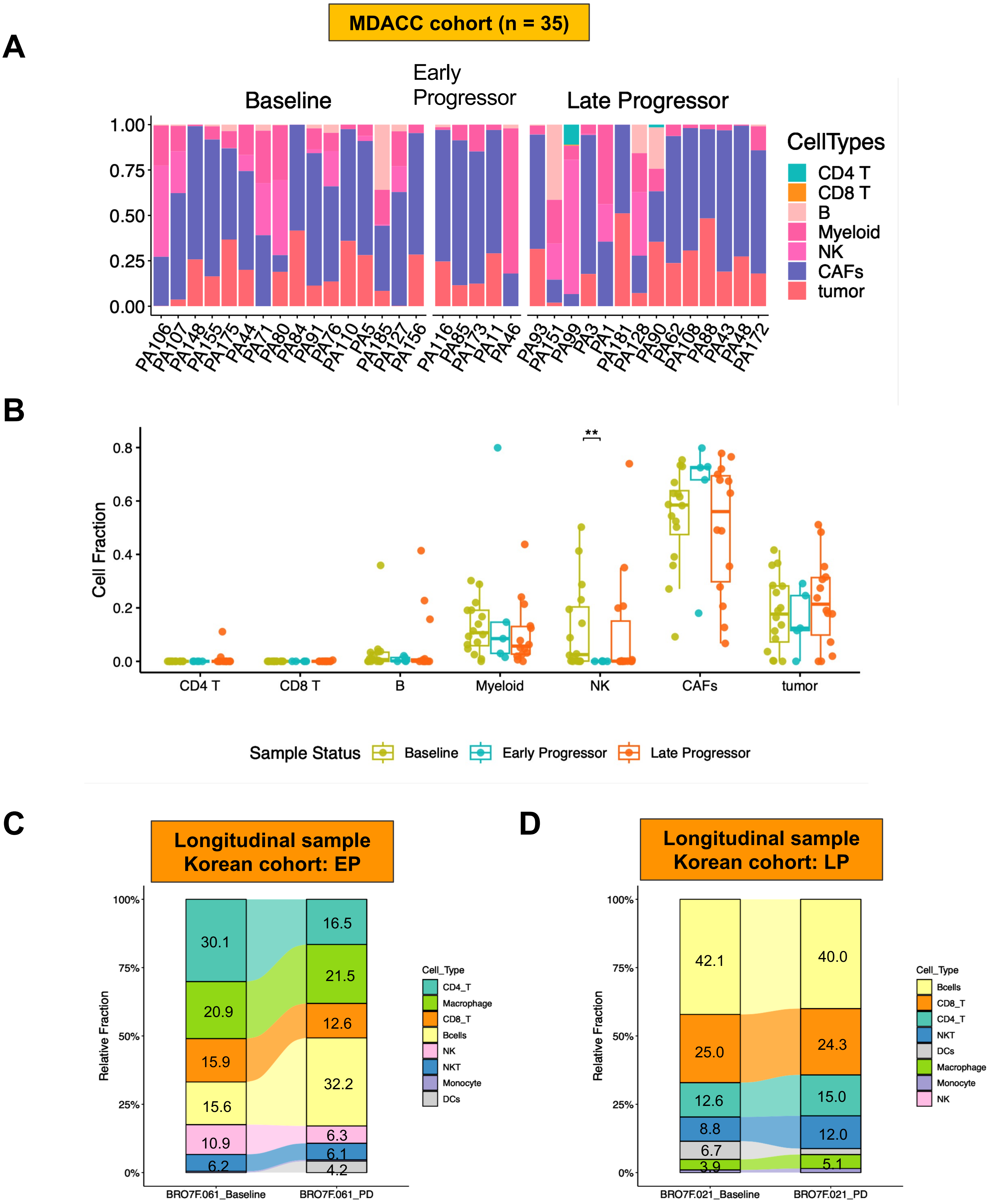
MDACC cohort (n = 35)
A
Baseline
Early Progressor
Late Progressor
1.00
0.75
0.50
0.25
0.00
PA106 PA107 PA148 PA155 PA175 PA44 PA71 PA80 PA84 PA91 PA76 PA110 PA5 PA185 PA127 PA156
PA116 PA85 PA173 PA11 PA46
PA93 PA151 PA99 PA3 PA1 PA181 PA128 PA90 PA62 PA108 PA88 PA43 PA48 PA172
CellTypes
CD4 T
CD8 T
B
Myeloid
NK
CAFs
tumor
B
Cell Fraction
0.8
0.6
0.4
0.2
0.0
**
CD4 T
CD8 T
B
Myeloid
NK
CAFs
tumor
Sample Status
Baseline
Early Progressor
Late Progressor
C
Longitudinal sample Korean cohort: EP
Relative Fraction
100%
75%
50%
25%
0%
30.1
20.9
15.9
15.6
10.9
6.2
16.5
21.5
12.6
32.2
6.3
6.1
4.2
BRO7F.061_Baseline
BRO7F.061_PD
Cell_Type
CD4_T
Macrophage
CD8_T
Bcells
NK
NKT
Monocyte
DCs
D
Longitudinal sample Korean cohort: LP
Relative Fraction
100%
75%
50%
25%
0%
42.1
25.0
12.6
8.8
6.7
3.9
40.0
24.3
15.0
12.0
5.1
BRO7F.021_Baseline
BRO7F.021_PD
Cell_Type
Bcells
CD8_T
CD4_T
NKT
DCs
Macrophage
Monocyte
NK
